# Invariant Feature Matching for Image Registration Application Based on New Dissimilarity of Spatial Features

**DOI:** 10.1371/journal.pone.0149710

**Published:** 2016-03-17

**Authors:** Seyed Mostafa Mousavi Kahaki, Md Jan Nordin, Amir H. Ashtari, Sophia J. Zahra

**Affiliations:** Center for Artificial Intelligence Technology, Faculty of Information Science and Technology, National University of Malaysia (UKM), Bangi, Selangor, Malaysia; Universitat de Valencia, SPAIN

## Abstract

An invariant feature matching method is proposed as a spatially invariant feature matching approach. Deformation effects, such as affine and homography, change the local information within the image and can result in ambiguous local information pertaining to image points. New method based on dissimilarity values, which measures the dissimilarity of the features through the path based on Eigenvector properties, is proposed. Evidence shows that existing matching techniques using similarity metrics—such as normalized cross-correlation, squared sum of intensity differences and correlation coefficient—are insufficient for achieving adequate results under different image deformations. Thus, new descriptor’s similarity metrics based on normalized Eigenvector correlation and signal directional differences, which are robust under local variation of the image information, are proposed to establish an efficient feature matching technique. The method proposed in this study measures the dissimilarity in the signal frequency along the path between two features. Moreover, these dissimilarity values are accumulated in a 2D dissimilarity space, allowing accurate corresponding features to be extracted based on the cumulative space using a voting strategy. This method can be used in image registration applications, as it overcomes the limitations of the existing approaches. The output results demonstrate that the proposed technique outperforms the other methods when evaluated using a standard dataset, in terms of precision-recall and corner correspondence.

## Introduction

Extraction of accurate and efficient correspondence features between different images is an important aspect of image processing and computer vision fields [1]. Furthermore, feature-based correspondence extraction techniques are more reliable and are typically computationally inexpensive [2]. The aim of this research study is to provide a robust method for extracting feature points in order to identify the corresponding areas in both the original and the target images. This method can be used in image processing applications, as it is capable of overcoming the limitations of the existing approaches. Therefore, an efficient and robust technique aimed at achieving accurate results in the matching step can enable the applications to produce more accurate results. Feature detection and matching are fundamental steps in many computer vision and image analysis applications, such as image matching and comparison [2], stereo matching [3], panoramic image stitching [4], scenic image registration [5, 6], and exemplary retrieval [7]. According to the extant literature, feature matching performance is closely linked to the information output from the correspondence extraction method. In this approach, feature matching techniques are affected by three main challenges [8], namely: (1) Different number of feature points may be extracted in the source and target images because of presence of noise or local variations in the images; (2) Feature points may be missed in the target image because of nose or occlusion variation; and (3) Local information pertaining to the features may change in different image scales and transformations, which can directly affect the feature matching results [9]. While different techniques for mitigating these limitations have been proposed, none is capable of eliminating them [10]. Therefore, a new feature matching technique, which is independent and can be generalized to all feature extractor techniques irrespective of the feature detection method employed, is required. In this study, two feature point sets, extracted from the source and target images obtained from extant work [9], are assumed to be available as inputs.

## Related work

Review of the pertinent related works indicates that feature correspondence performance is strongly dependent on the information produced in the feature extraction steps. Hence, an independent feature matching method should be able to generalize the algorithm to all interest point approaches. However, most available interest point matching approaches require significant number of points to be matched in order to yield acceptable results. The local information pertaining to the feature points is usually insufficient to extract the correspondence information [11]. Consequently, additional information pertaining to the feature points, such as geometric or spatial data, could be included, as it has potential to extract more accurate correspondence information. According to the extant literature, currently available feature matching methods are divided into three main categories, namely graph-based matching, local matching, and geometric-based matching methods.

### Graph-based matching

Presently available feature matching techniques based on graphs interpret the extraction of correspondence points as a graph matching problem and thus require an algorithm to estimate the results [11]. The corresponding problem in graph matching can be formulated by either spectral methods or through integer quadratic programming (IQP), whereby the former approach is based on Eigen decomposition of the neighborhood matrices. It was introduced by Umeyama [12], who used it to determine the permutation matrix. Shortly after, Shapiro and Brady [13] proposed their technique to extract the correspondence features in different images by minimizing the Euclidean distance of row values in the modal matrices. As this method is sensitive to false positives and different modalities [11], it is not robust enough to produce a sufficient matching accuracy in a complex scene. Thus, a compromise between performance and computational complexity based on the spectral matching technique was later proposed [14]. Some authors chose to formulate the matching problem as an Integer Quadric Program (IQP) [15] to estimate the result by solving the optimization problem [16]. A new assignment approach was used to extract the correspondence features iteratively among attribute graphs [17]. While this method is sufficiently robust to extract the correspondence features, it is inefficient when applied to images containing complex information.

### Local matching

Some researchers consider the information pertaining to the point neighborhood to develop their method for extracting the final correspondence or the candidates. This category of matching techniques is also known as neighborhood-based methods. Based on the extant literature, neighborhood-based matching methods can be divided into three subcategories: Threshold-based, Nearest-Neighbor (NN)-based and Nearest-Neighbor-Distance-Ratio (NNDR)-based [18]. In the approaches belonging to the first category, if the Euclidean distance between the features is below a predefined threshold value, the features are considered matched. This method is unreliable, as a single feature in the first image may have several correspondence points in the second image. To address this problem, in the Nearest-Neighbor approach, the nearest neighborhood and the distance must be below a predefined threshold to consider the feature points as matched points. However, this may result in many false positives (FPs). Fischker and Bolles [19] developed a new method that can remove the outliers produced by the NN method by using random sample consensus (RANSAC). In this method, known as Nearest-Neighbor-Distance-Ratio (NNDR), a predefined threshold value is considered between extracted features in the source and target images, while the NN method is also applied. In this method, duplicate extracted features are considered as outliers and the features can only have one correspondence point in the target image [11]. However, while higher performance with respect to extracting the correspondence points is achieved by using RANSAC technique, this approach suffers from higher computational cost.

Empirical evidence indicates that, even though local matching techniques achieve higher performance compared to that of graph-based approaches, repeated features may be ignored and false positive correspondence extraction is still possible [18]. In this paper, a combination of neighborhood methods and geometric approaches is proposed as a means of overcoming these drawbacks.

### Geometric-based matching

Since the concept of geometric-based matching was proposed by Lamdan and Wolfson [20] in 1988, many other matching methods have been developed in this category. Geometric-based matching methods extract the spatial information pertaining to the feature points to identify the correspondence points in different images and thus achieve more reliable results relative to other methods [8]. Given that extracting the spatial information among the feature points is computationally expensive and higher NP-hard, these shortcomings have been the subject of extensive studies. Moreover, geometric-based matching methods require high number of feature candidates to estimate the transformation matrix. When the number of features to match is excessively high, this can adversely affect the computational complexity of the algorithm. In this case, a non-iterative feature matching method is required to reduce the response time. Moreover, the correspondence point extraction does not require estimating the transformation parameter in the first phase, as this can be performed after matching the features [11]. Extracting the correspondences based on finding the scale and orientation is not computationally efficient even when these tasks are performed on pairs of features [11]. A new efficient geometric-based method that employs neighborhood candidate extraction and geometric correspondence extraction has been proposed by Hu and Ahuja [21]. According to Yoon and Kweon [22], in this technique, a small number of correct correspondence features are extracted while filtering the outliers. You et al. [23] proposed a feature matching technique based on Hausdorff distance to measure the similarity of feature points. Their method is sensitive to outliers, as well as computationally expensive, and is not invariant to geometric transformation. Taejung and Yong-jo [24] introduced a new dissimilarity metric based on the corner strength and transformation estimation, following the previous work of Jung and Lacroix [25]. Their method is inefficient, as it requires matching of several groups of features. To overcome this limitation, Zhou et al. [26] proposed Delaunay triangulation (DT) technique as a means of extracting correspondence features. The interior DT angle values are measured to identify the matched points in different images [27]. Their method is based on the triangle formed by the feature points, whereby the angles are measured to extract the correspondence points. While this method is based on interior angles and the local structure of the triangles, it is reliable under image translation and is highly robust in noisy environments [26]. However, it would fail under high geometric transformation in which the DT angles are not identical [8]. Affine-length and triangular area (ALTA) that is invariant to geometric transformation was introduced by Awrangjeb and Lu [8] to estimate the correspondence points [8]. The curvature and affine-length values of the contour are measured in their approach to extract the initially matched candidates. This allows an imaginary triangle to be defined for each combination of three candidates, and this curvature information of the corner points is used to find the matched points. Available data indicate that this method is not reliable and it is dependent on the feature detection method employed, as the curvature information of the corner points is measured, which can vary in different images, especially those affected by high deformation.

In this paper, a new correspondence extraction based on triple-wise dissimilarity measure technique is proposed, which uses only the coordinate outputs of the feature detector method. In this method, extracted information of the path between two specific features in source and target images is accumulated in a 2-D space as a means of identifying the correspondence points. This method is robust and invariant to different image transformations. Moreover, the proposed feature matching method has been used as a part of image registration technique to demonstrate its robustness in real application. Finally, to evaluate the proposed method, different feature matching and image assessment evaluations were performed using several comparison criteria of well-known algorithms.

## Spatially Invariant Feature Matching

Some existing feature matching methods use local feature information, such as curvature values or other information pertaining to the interest point, to provide correct output [28, 29]. These dependencies limit the feature matching algorithms to the specific task and cannot be generalized to cases in which the output information of different detectors varies. The spatial invariant feature matching (SIFM) method is generalized for all detection techniques as it only considers the feature coordinates output by any feature point detector. Moreover, as it is based on invariant feature dissimilarity techniques, the proposed method is also invariant to local and global deformations. In this section, SIFM method based on the geometric invariant theory is introduced [30].

### Preparations and formulation

**Fig 1:**
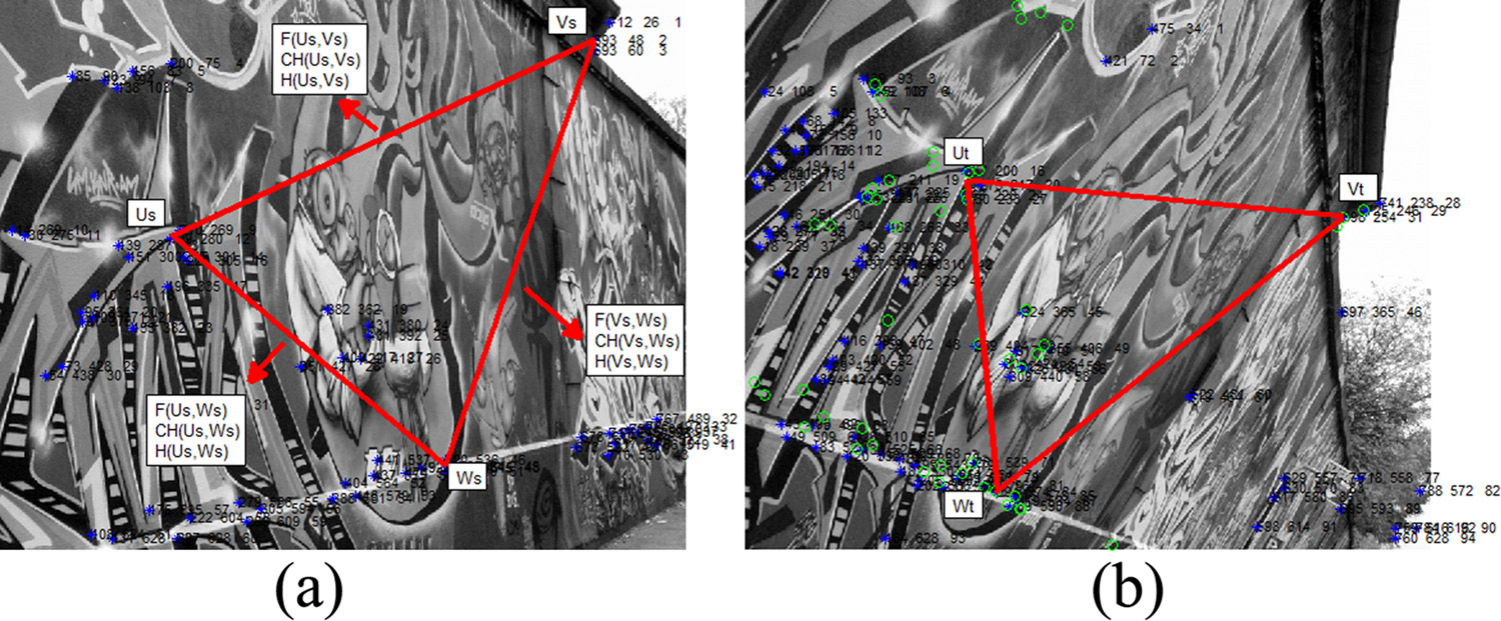
Candidate extraction on graffiti image [32]. (a) The source image; (b) The target image.

Denoting the feature points from the source image (*S*) as *S*(*x*,*y*) and the feature points from the target image (*T*) as *T*(*x*,*y*), the general definition for extracting the transformation between two images can be defined by Eq (1). According to the extant literature [30, 31], at least three feature points are required, as presented in Fig 1, to extract the six unknown parameters (*a*,*b*,*c*,*d*,*t*_*x*_ *and t*_*y*_) in transformation definition in Eq (1).

$$S(x,y)\to T(x,y),\left[\begin{array}{c} x_{t}\\ y_{t}\\ 1 \end{array}\right]=\left[\begin{array}{ccc} a & b & \begin{array}{c} t_{x} \end{array}\\ c & d & t_{y}\\ 0 & 0 & 1 \end{array}\right]\left[\begin{array}{c} x_{s}\\ y_{s}\\ 1 \end{array}\right],$$ (1)

where *a*,*b*,*c* and *d* support the image rotation, image reflection, and projective transformations, while *t*_*x*_ and *t*_*y*_ support image translation. Hence, the primary issue in correspondence extraction is to determine the six unknown variables in Eq (1). Extraction of the most accurate correspondence features in the source and target images can satisfy the initial parameters, which can yield values of the six unknown variables. The main aim of this work is to extract the most accurate correspondence features in both source and target images, which is achieved by adopting the proposed invariant feature matching method described in the next section. In the first step, the initial candidate correspondence features are extracted from the source and target image by using invariant dissimilarity metric. In the next step, for each three points in the source image, three initial similar features are considered in the target image randomly. Then, in each iteration, a combination of three similar triangle features is compared in both source and target images to estimate the unknown variables in Eq (1). Finally, in the last step, a voting method is performed to extract the most accurate invariant correspondence features from the candidate set. In this step, the frequency, intensity histogram and color histogram of the line between triple features are measured to ensure robustness by removing the outliers. Eventually, the best fit of three features in both source and target images is achieved to calculate the transformation information, which allows matching all the other features in different images.

### Interest points extraction

Generally, in feature extraction methods, candidate feature points are extracted based on the similarity values of neighbor information. However, a combination of noise and deformation of imagery can affects the performance in these techniques. To overcome this issue, the method proposed in this study extracts the candidate features based on a triangle structure similarity instead of using only the neighbor information of the pixels. In this method, robustness is increased by utilizing the spatial information of a selected path between the features. More specifically, the approach is based on the assumption that *P*_*s*_ = [*u*_*s*_, *v*_*s*_, *w*_*s*_] pertains to the feature points extracted from the source image (*S*), whereas *P*_*t*_ = [*u*_*t*_, *v*_*t*_, *w*_*t*_] relates to the feature points extracted from the target image (*T*). Fig 1 presents an example of triangle structure between two different images sourced from the Featurespace dataset [32]. It is important to extract the most accurate triangle candidates in the source and target images that are most similar. To achieve this objective, three randomly selected features from source image are selected to compare to the initial candidates from the target image. In the next step, the dissimilarity result of this triple-wise method is accumulated in a 2D similarity space, which stores the information for all extracted triangles.

According to Fig 1, considering the line vector $\hat{\text{L}}$ between two randomly selected feature points in the image, for example *v*_*s*_ and *w*_*s*_, ${\hat{L}}_{{\text{v}}_{\text{s}},{\text{w}}_{\text{s}}}$ is defined as:

$${\hat{L}}_{{\text{v}}_{\text{s}},{\text{w}}_{\text{s}}}=\delta_{{\text{v}}_{\text{s}},{\text{w}}_{\text{s}}}\text{exp}(j\theta_{{\text{v}}_{\text{s}},{\text{w}}_{\text{s}}}\;),$$ (2)

where $\delta_{{\text{v}}_{\text{s}},{\text{w}}_{\text{s}}}$ denotes the length and θ represents the orientation of the line ${\hat{L}}_{{\text{v}}_{\text{s}},{\text{w}}_{\text{s}}}$. Using this approach allows $\hat{\text{L}}$ to be defined for each feature point pair. Given $\left\{\hat{\text{L}}\right\}$, denoting the discrete image values with *i* grey levels, the probability that a pixel of level *i* would be located on the line $\hat{\text{L}}$ is given by:

$$p_{\hat{L}}\left(i\right)=p\left(L=i\right)=\frac{n_{i}}{n}\;,0\leq i\leq \delta -1,$$ (3)

where *n*_*i*_ represents the number of similar intensity, while *n* denotes the total number of pixels. Given the above, the cumulative distribution function can be calculated using the following expression:

$$cdf_{\hat{L}}=\sum_{j=0}^{i}p_{\hat{L}}\left(j\right)\;,\;0\leq i\leq \delta -1,$$ (4)

which is the normalized feature across the line. The result yielded by $\text{y}=\text{T}(\hat{\text{L}})$ can be further transformed into a flat feature vector {y} by calculating the linearized *cdf* across the available value range. This transformation can be expressed as:

$$y=T\left(k\right)=cdf_{\hat{L}}(k),$$ (5)

where k should be within the [0,*δ*] range, whereas *T* produces the transform in the normalized range. As a result, the transform obtained above can be applied to the feature values using the expression:

$$y^{\prime }=y.\left(\text{max}\left\{\hat{L}\right\}-\text{min}\left\{\hat{L}\right\}\right)+\text{min}\{\hat{L}\}.$$ (6)

As the last invariant feature of the distinct path between features, the frequency of the line $\hat{\text{L}}$ is extracted. However, while the information extraction of the features is a pairwise problem, the final dissimilarity comparison of the candidate features is a triple-wise problem. Thus, the frequency values can be measures by using the expression below:

$$f^{\prime }=\sum_{j=0}^{i}\hat{L}(i)\;,\;0\leq i\leq \delta -1,$$ (7)

where *f*′ is the line information extracted from the vector $\hat{\text{L}}$. The frequency value in a binary line $\hat{\text{L}}$ is simply defined as the number of changes from white to black or vice versa [33]. The dissimilarity between features is calculated by using the normalized eigenvector correlation (NEC) and signal directional differences (SDD) techniques, as described in the next section. Based on a tradeoff between performance and efficiency, the width of the extracted line between two features can be adjusted.

### NEC dissimilarity metric

Normalized eigenvector correlation is a new invariant dissimilarity metric, which is proposed based on information theory. Invariant vector dissimilarity is required to ensure that a dissimilarity metric is invariant. Based on the available experimental evidence, Von Neumann entropy *S*(*ρ*) [34] is a good candidate, as it is invariant under different changes [35, 36]. Considering

$$S\left(\rho \right)=S(U\rho U^{†})$$ (8)

where *U* denotes unitary transformation, this entropy depends on the eigenvalues of the density matrix (*ρ*) only, which can be defined as:

$$S\left(\rho \right)=-Tr\rho \;\text{ln}\;\rho =-\sum_{1}^{N}\lambda_{i}\;\text{ln}\;\lambda_{i},$$ (9)

where *λ*_*i*_ are the eigenvalues of the density matrix *ρ*, and *N* is the number of elements in *ρ*. In order to evaluate the dissimilarity matrix, for each feature vector ${\vec{v}}_{i}$ in the source and target images, the *S*(*ρ*) value is calculated and accumulated in an *l* × *m* probability matrix defined as:

$$p={\left[\begin{array}{ccc} D_{Ent_{1,1}} & \ldots & D_{Ent_{1,m}}\\ \vdots & \ddots & \;\\ D_{Ent_{l,1}} & \; & D_{Ent_{l,m}} \end{array}\right]}_{l\times m},$$ (10)

where *D*_*Ent*_ can be expressed in terms of similarity distances between two features:

$$Sim\left(S_{l},T_{m}\right)=D_{Ent_{l,m}}=\sqrt{S{\left(\rho_{T}\right)}^{2}-S{\left(\rho_{S}\right)}^{2}}.$$ (11)

where *Sim*(*S*_*l*_, *T*_*m*_) calculates the entropy distance between two features in the source and target images. Each row *l* and column *m* of the probability matrix *p* indicates the dissimilarity value between features *S*_*l*_ from the source image and *T*_*m*_ from the target image. Consequently, the minimum dissimilarity in each row and column of *p* indicates the maximum similarity of the features. Therefore, the similarity matrix provides a ranking of the dissimilarity indices for all features in the source and target images.

Based on the characteristic vector properties, in a transformation *T* where $T:R^{n}\to R^{n}$, the vector $\vec{v}$ that has the form of $T(\vec{v})=\lambda \vec{v}$ is only scaled by *λ*. In this transformation, the vector $\vec{v}$ is called the eigenvector, and the corresponding *λ* values associated with them are referred to as eigenvalues. Correlation-based dissimilarity measurement metric known as NEC is proposed based on these properties of the eigenvectors in Von Neumann entropy. The NEC is defined as:

NEC=1N∑i=1m∑i=1nhsvslnhtvt, (12)

where *h*_*s*_ and *h*_*t*_ are calculated based on a normalized histogram of distances, and *v*_*s*_ and *v*_*t*_ are calculated based on normalization of the eigenvectors and eigenvalues, as defined below:

$$\left\{ \begin{array}{l} h_{s}=\frac{hist\left(S\right)}{D_{s}K},D_{s}=dist\left(\text{min}\left(S\right),\text{max}\left(S\right)\right)\\ h_{t}=\frac{hist\left(T\right)}{D_{t}K},D_{t}=dist\left(\text{min}\left(T\right),\text{max}\left(T\right)\right) \end{array}\right.,\{\begin{array}{l} v_{s}=\frac{arg\;\underset{s}{\text{max}}({\vec{v}}_{s})}{\lambda_{s}}\\ v_{t}=\frac{arg\;\underset{t}{\text{max}}({\vec{v}}_{t})}{\lambda_{t}} \end{array},$$ (13)

where ${\vec{v}}_{s}$ and ${\vec{v}}_{t}$ are the eigenvectors, while *λ*_*s*_ and *λ*_*t*_ are the corresponding eigenvalues extracted from the source set *S* and the target set *T*, respectively. The *K* value is a normalized factor extracted from the mean of all neighboring intensity values in both the source and target images, which can be defined as:

$$K=\frac{1}{N}\sum_{i=1}^{m}\sum_{j=1}^{n}k_{i,j},k_{i,j}\in S,T.$$ (14)

### SDD dissimilarity metric

In the currently available dissimilarity metrics using the raw image data to measure dissimilarity, the data is not subjected to any processes, such as information extraction, and is obtained directly from the source. Since gray-level values may be affected by different sources, as well as shifts in the signal caused by different lighting conditions, extracting the distances between these values for similarity measurement is insufficient. Moreover, while a signal from an image can take any value in (-∞, +∞), only its active part is important. The part of the signal that contains non-zero values that exceed a predefined threshold is considered active. An image signal may be shifted due to different lighting conditions or because different sources are used when capturing the signal. As the existing dissimilarity metrics fail to calculate the image similarity under these conditions, to overcome this problem, Signal Directional Differences (SDD) is proposed. The goal is to support the NEC metric in identifying the features with minimum dissimilarity. In order to calculate the SDD vector for a signal, in each step, the differences in the signals are calculated. These differences can have zero, negative or positive values. SDD can be expressed as:

$$SDD=\sum_{i=1}^{l}\frac{\left|S_{i}-S_{i-1}\right|}{\sigma_{1}^{2}}-\frac{\left|T_{i}-T_{i-1}\right|}{\sigma_{2}^{2}},$$ (15)

where *l* denotes the maximum length of the first signal *S* and the second signal *T*, and *σ* is the standard deviation in the signal values, given by:

$$\sigma =\sqrt{\frac{1}{N}\sum_{i=1}^{N}{\left(x_{i}-\mu \right)}^{2}}.$$ (16)

In the expression above, *μ* is the mean of all the values and is defined as:

$$\mu =\frac{1}{N}\sum_{i=1}^{N}(x_{i}).$$ (17)

In similar images, the values of the signal steps are also similar. Consequently, the difference between the values remains the same. Thus, the starting point for the calculations performed in the SDD technique corresponds to the starting point of the active part of the signal. This helps avoid performing unnecessary calculations that would involve inactive parts of the signal. Moreover, the standard deviation for each signal helps normalize the values, while showing the amount of variation from the mean. The variation in the SDD is within the [0,+∞] range, whereby lower value indicates greater similarity in the input images. As shown in Eq (13), the SDD is a vector of differences between two signals. Thus, the sum of the SDD values is used to measure the dissimilarity between two image blocks.

### SIFM algorithm

The main objective of SIFM is to extract the most accurate three correspondence points in the source image and the target image, which is achieved using the proposed dissimilarity metrics. In this method, two interest point sets, source *S* and target *T*, are assumed to be available, as the inputs are extracted from the source and target images, respectively. The most accurate correspondence points in *T* and *S* can be extracted by aiming to obtain the highest similarity values in the lines between the points. Thus, to meet this objective, two pairs of interest points from the source image are selected randomly and are compared to all point pairs in the target image using the line features between them. Dissimilarity between features in the source and target sets is measured using the proposed SDD and NEC metrics. If the similarity value is satisfied, then the line is considered to be the first line candidate; otherwise, the next point pair in the target image will be compared until the best fit for the line is selected. Algorithm 1 presents the feature matching to find the candidates. Three features, including color histogram, intensity histogram and frequency from two interest points, are extracted by drawing a line between them. Let us assume that *L*(*v*_*s*_,*u*_*s*_) in the source image is positioned along the extracted line from *v*_*s*_ to *u*_*s*_. In the first step, using the NEC and SDD dissimilarity metrics, SIFM seeks all available lines between points in the target image to find the lines most similar to *L*(*v*_*s*_,*u*_*s*_). There is a possibility of finding more than one similar line in the target image. In that case, the real candidate is confirmed by matching the third point. The process resumes by finding the third point *w*_*t*_ in the target image that meets the the triangle similarity condition based on the extracted line features. This aim is achieved using a dissimilarity comparison by finding the best similarity for *L*(*v*_*s*_, *w*_*s*_) and *L*(*v*_*t*_, *w*_*t*_) for which *L*(*u*_*s*_, *w*_*s*_) and *L*(*u*_*t*_, *w*_*t*_) are the most similar as well. This results in obtaining three similar points, *P*_*s*_ = [*u*_*s*_, *v*_*s*_, *w*_*s*_] and *P*_*t*_ = [*u*_*t*_, *v*_*t*_, *w*_*t*_]. Several candidate points may be extracted based on the iteration presented in Algorithm 1. Thus, the final matched features set is a subset of all extracted features using Algorithm 1. In the next step, the final correspondence feature set is extracted based on the voting algorithm presented in Algorithm 2, which uses the cumulative 2D dissimilarity space. The highest dissimilarity index is achieved using Algorithm 2, which extracts the most accurately matched features. The proposed feature matching method includes the following steps: (1) three feature points are selected from the source image to form a triangle structure; (2) according to Algorithm 1, the most similar triangle in the target image is extracted using proposed SDD and NEC methods; (3) a voting method presented in Algorithm 2 is used to determine the most accurate correspondence features points; (4) the six unknown variables in Eq (1) are calculated to estimate the transformation matrix and determine the transformation matrix using the most accurate correspondence points; (5) all feature points from source image are transformed to the target image by using transformation matrix; and (6) the inverse transform is calculated before reconstructing the target image to the source image for image registration purpose. In this method, the feature points that are not detected in the target image can be predicted by using transformation matrix, which can be used in the transform image identification application [8]. In Algorithm 1, two input images, including two sets of points as source image points *S* = {*s*_1_,*s*_2_,…,*s*_*i*_} and target image points *T* = {*t*_1_,*t*_2_,…,*t*_*j*_}, are assumed to be available. The result of this process are the correspondence sets $Q_{t}={\left\{(x_{it},y_{it})\right\}}_{i=1}^{n}$ and $Q_{s}={\left\{(x_{is},y_{is})\right\}}_{i=1}^{n}$ from target and source images, respectively. A voting strategy, presented as Algorithm 2, based on point candidates is implemented to extract the most accurate correspondence points from the candidate points obtained in the preceding step via Algorithm 1. The inputs comprise of correspondence sets $Q_{t}={\left\{(x_{it},y_{it})\right\}}_{i=1}^{n}$ and $Q_{s}={\left\{(x_{is},y_{is})\right\}}_{i=1}^{n}$ from target and source images, respectively, and the output provides the best set of correspondence points, defined as *T*_*r*_.

**Algorithm 1.** Feature matching

for (*i* = 1;*i* ≤ *P*;*i*++){

    *s*_*τ*1_,*s*_*τ*2_,*s*_*τ*3_ = *Random*{*s*_1_,*s*_2_,…,*s*_*i*_};

    *F*_1_ = Extract features in {*s*_*τ*1_,*s*_*τ*2_};

    for (*i* = 1;*i* ≤ *M*;*i*++){

        for (*j* = 1;*j* ≤ *M*;*j*++){

            *TF*_1_ = Extract feature in {*t*_*i*_,*t*_*j*_};

            if (*NEC*, *SDD*(*TF*_1_,*F*_1_)<*T*) {

                for (*k* = 1;*k* ≤ *M*;*k*++){

                    *TF*_2_ = Find feature between {*t*_*i*_,*t*_*k*_};

                    *F*_2_ = Find feature between {*s*_*τ*2_,*s*_*τ*3_};

                    if (*NEC*, *SDD*(*TF*_2_,*F*_2_)<*T*){

                        *TF*_3_ = Extract feature between {*t*_*k*_,*t*_*j*_}

                        *F*_3_ = Extract feature between {*s*_*τ*3_,*s*_*τ*2_};

                        if(*NEC*, *SDD*(*TF*_1_,*F*_1_)<*T*){

                            *Q*_*ϕ*_ = {*t*_*i*_,*t*_*j*_,*t*_*k*_};

                            *Q*_*sϕ*_ = {*s*_*τ*1_,*s*_*τ*2_,*s*_*τ*3_};

                            *ϕ*++;

                        }

                    }

                }

            }

        }

    }

}

**Algorithm 2.** Voting method

for (*i* = 1;*i* ≤ *ϕ*;*i*++){

          *TR*_*i*_ = ∑ *NEC*,*SDD*(*Qs*_*i*_) *in Q*;

}

*T*_*r*_ = *Max*(*TR*);

**Notes:**

By default

*P* = 10, *TF*_1_ ≅ *F*_1_, *TF*_2_ ≅ *F*_2_, *TF*_3_ ≅ *F*_3_. *ϕ* is the counter for results.

*M* is the number of the target interest points.

ϕ=length(Q).

Features between two points {*s*_1_, *s*_2_} are: *L*_*s*1_ = *Line*{*s*_1_ → *s*_2_}, *F*_int1_ = *Hist*(*L*_*s*1_),

$$F_{color1}=colorHist(L_{s1}),\;F_{frequency1}=count(Edges(L_{s1})),\;F1=\{F_{\text{int}1},F_{color1},F_{frequency1}\}.$$

where *P* is the number of iterations for finding the matching points. Higher *P* values allow the algorithm to achieve greater accuracy and provide voting strategy with sufficient input points and parameters to calculate the final output.

## Experimental Results

**Fig 2:**
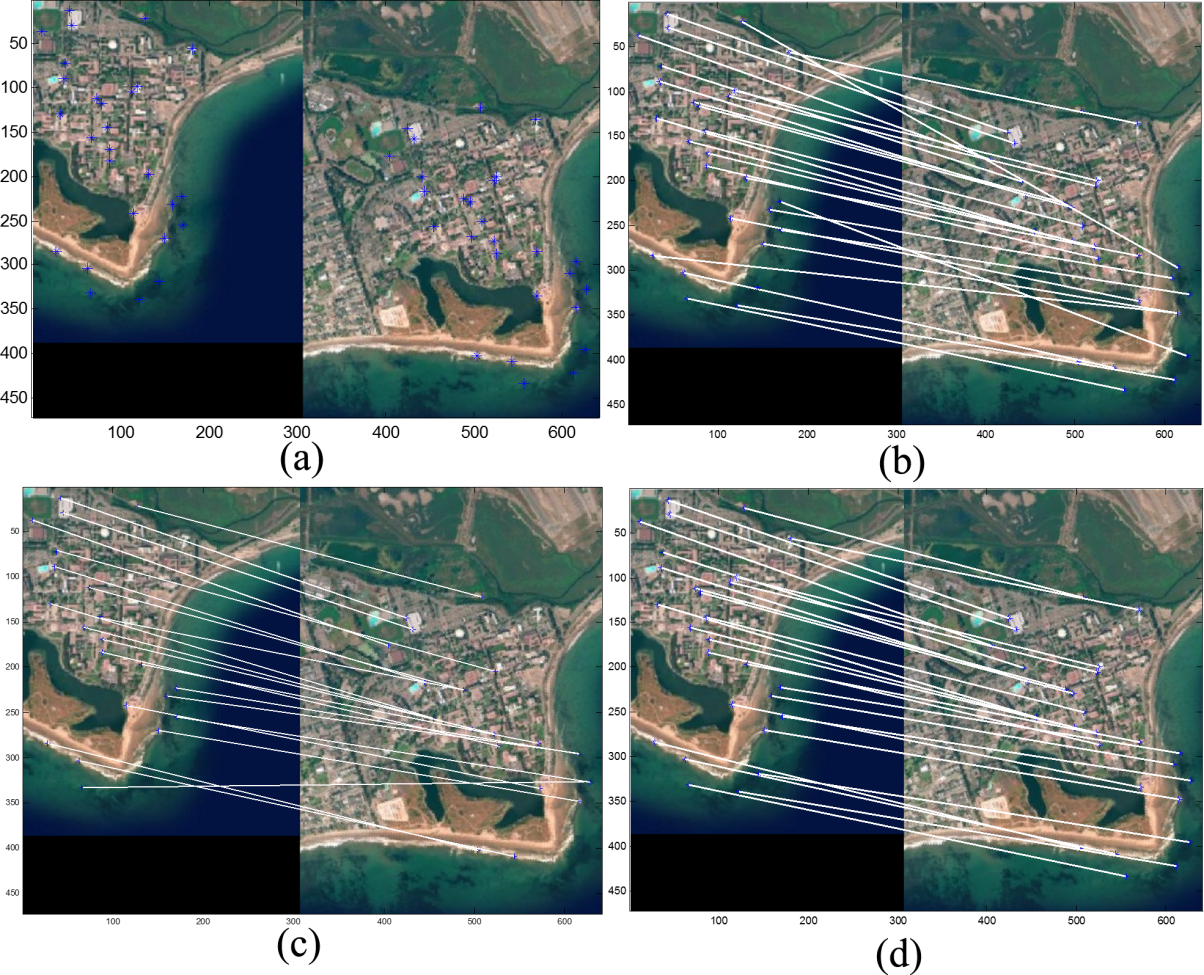
Corner matching comparison under viewpoint change of high resolution IKONOS satellite image (UC Santa Barbara) [40]. (a) Detected points; (b) DT matching method [41]; (c) ALTA matching method [8]; (d) SIFM matching method.

In this section, corner matching evaluation and analysis of the results is presented. To evaluate the results, corner correspondence (CC) [10] and precision-recall [37], both of which are standard evaluation techniques, are used to demonstrate the robustness of the proposed corner matching technique in comparison with other currently used and well-known approaches. To evaluate its performance, the proposed method is compared with two approaches, namely DT [38] and ALTA [8], because they are the most promising methods among the available matching techniques and exhibit superior performance relative to other techniques [8]. To evaluate the proposed method, the SIFMDB [39] dataset is used (data in S1 Dataset). A total of 152 test images—47 original images and 105 transformed images—are used to evaluate the aforementioned corner matching techniques. To develop appropriate evaluation criteria, various types of images—such as aerial imagery with different viewpoints, scene imagery with different illumination, and artificial standard images—are included in the dataset. In order to evaluate the proposed method, different transformation effects are used for assessment, including:

thirteen rotated images, obtained by changing the angle θ in the [−90°, +90°] range, at 15° increments; seven scaled images, obtained by changing the uniform scale factor *S*_*x*_ = *S*_*y*_ in the [0.4,1.6] range, at 0.2 increments; combined transformations, including rotation and scale transform with different rotations θ in [−20°, +20°] at 5° steps, and scale factors *S*_*x*_, *S*_*y*_ in the [0.4,1.6] range, at 0.2 increments; and the occlusion effect in the [0%, 50%] range, with 5% steps. Fig 2 depicts the feature matching results obtained when a combination of different transformations of high resolution IKONOS satellite image (UC Santa Barbara) [40] is compared to other methods.

To evaluate the feature matching methods, repeatability score (CC) [8], precision and recall are chosen in order to measure the evaluation metrics results of different techniques. The repeatability score considers the number of correctly extracted correspondence features in the source and target images. The maximum repeatability score (CC = 1) indicates that the number of extracted points in the original image and the target image is identical and all the correspondence features are extracted correctly. This can be expressed as:

$$CC=\frac{N_{m}}{2}(\frac{1}{N_{s}}+\frac{1}{N_{t}}),$$ (18)

where *N*_*s*_ is the number of detected features in the source image, *N*_*t*_ is the number of detected features in the target image, and *N*_*m*_ is the number of matched features.

Another standard evaluation method employed to compare the results is average precision and recall, formulated as:

$$Precision=\frac{TP}{TP+FP}\;,\;Recall=\frac{TP}{TP+FN}\;,$$ (19)

where TP is the true positive or the number of correctly detected feature points, FP is the false positive or the number of incorrectly detected feature points (or unexpected results). The total number of detected feature points in both source and target images is closely related to the image resolution, image size and the transformation. Moreover, different feature extraction methods would result in a different number of extracted features depending on their specific algorithms. In corner matching, the aim is to extract the correspondence points based on the similarity values between the features. Therefore, the number of unexpected correspondence points, or FPs, is not high. Consequently, the precision score does not change significantly under different image effects. Therefore, to compare the results yielded by different techniques under different image transformations, average precision-recall known as F-measure [42] based on Eq (20) below is calculated.

$$F\_measure=\frac{2}{\frac{1}{Recall}+\frac{1}{Precision}}.$$ (20)

**Fig 3:**
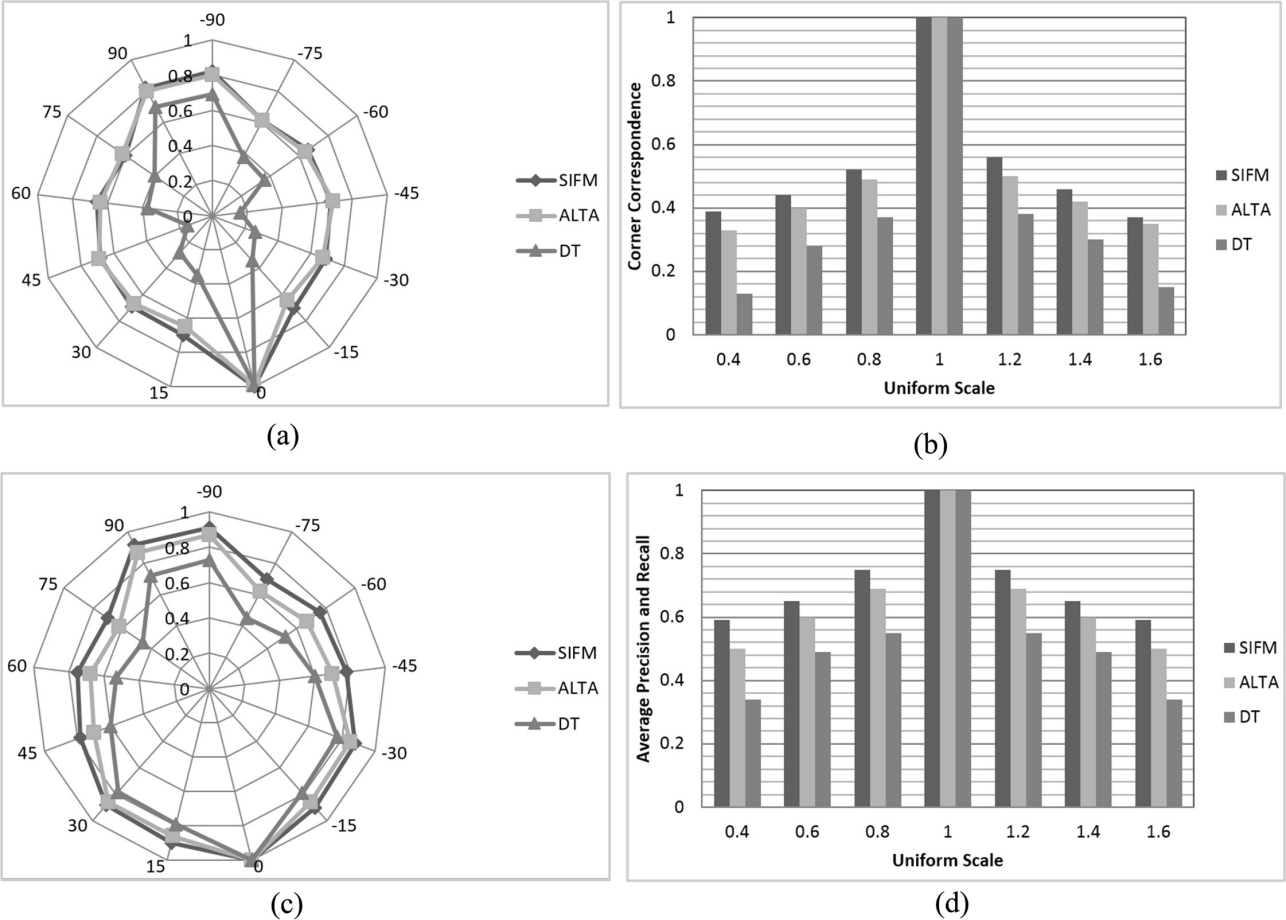
Matching results of different methods using SIFM dataset. (a) Average CC score for rotation at 13 different angles θ in [−90°, +90°], 15° apart; (b) Average CC score for uniform scale factors S ∈ [0.4,1.6], 0.2 apart; (c) F-measure results for Rotation at 13 different angles θ in [−90°, +90°], 15° apart; (d) F-measure results for Uniform scale factors S ∈ [0.4,1.6], 0.2 apart.

Fig 3 presents matching results of different methods using the SIFM dataset, including the average of CC and F-measure results under different image effects.

**Fig 4:**
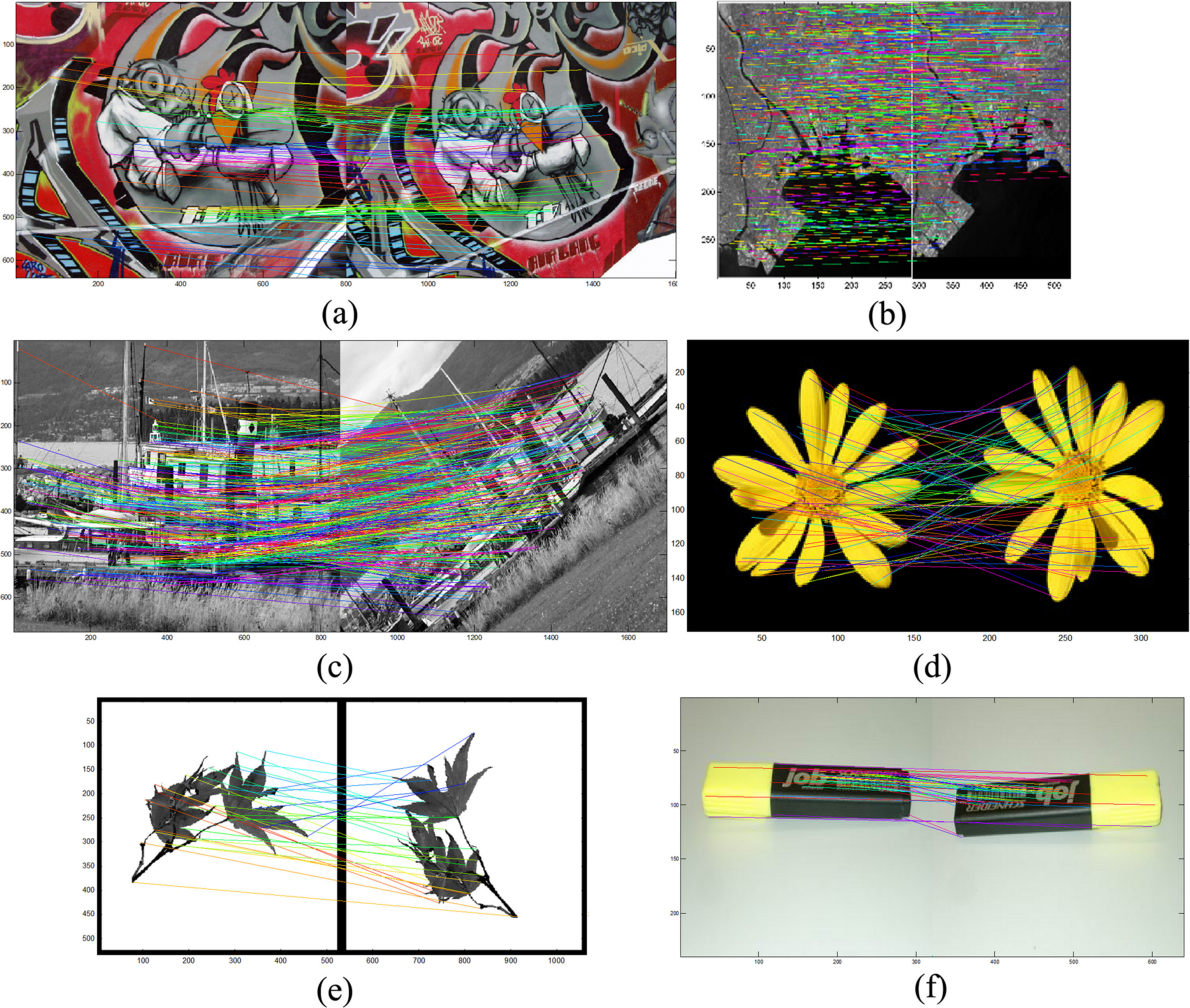
Results of the SIFM method for different images under different transformation effects. (a) Matching results of graffiti image; (b) Matching results of a satellite image at different viewpoints (Tokyo bay); (c) Matching results of boat image at different viewpoints; (d) Matching results of a flower image; (e) Matching results of a leaf image; (f) Matching results of a marker image.

It is not possible to automatically calculate the number of matched points, which is equivalent to the number of repeated points; therefore, calculation of the CC requires human visual inspection or ground truth. The point matching results obtained via the SIFM, ALTA and DT methods were compared in Fig 3(A) under different image rotations using the DLR method [43]. Under some rotation angles used in testing, the ALTA and SIFM methods yielded similar results; however, the DT method was less accurate. The corner correspondence results of various techniques under uniform scaling at seven different scales are presented in Fig 3(B). As can be seen, SIFM yields better results for scale changes, while ALTA is superior to the DT method. The comparison of results yielded by different techniques under transformation effects using F-measure are presented in Fig 3(C) and 3(D), while those pertaining to different image rotation at 13 different angles θ in [−90°, +90°] are depicted in Fig 3(A) and 3(C). The results achieved at 90° for all methods are better than those obtained for other angles because rotation of images in *iπ* radian remains more similar at that angle but not the other angles [43]. Changing the local information of the features at different angles can directly affect the feature matching results. Fig 4 presents the matching results obtained for different imagery types from the SIFMDB dataset, which indicate a high correspondence rate under different image deformations, such as different viewpoints and rotation.

**Fig 5:**
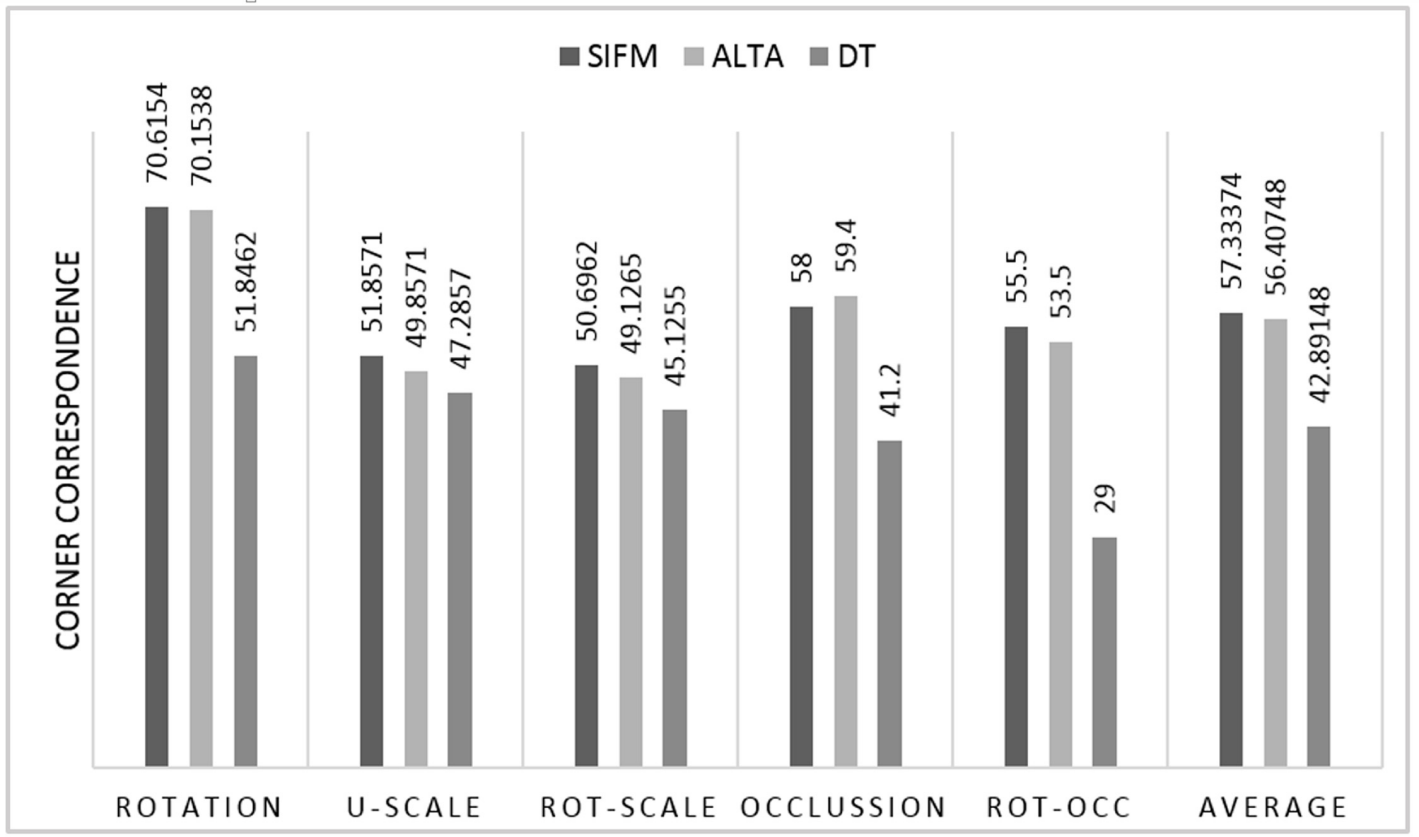
Corner correspondence results of Rotation, U-Scale (uniform-scale), Rot-Scale (rotation-scale), Occlusion, and Rot-Occ (rotation-occlusion).

Different types of images, including artificial, satellite, and natural imagery, have been used to show the results of the proposed method in Fig 4. The results of matching standard graffiti images from different viewpoints sourced from Featurespace standard dataset are presented in Fig 4(A), while Fig 4(B) pertains to a satellite image at different viewpoints. Standard boat image at different viewpoints is used to show the results of point matching method in Fig 4(C). Fig 4D–4F present the results achieved by the matching technique using different transformations. Fig 5 shows the overall CC results yielded by the SIFM, ALTA and DT matching methods under different geometric transformations. As can be seen, the SIFM method provided a higher mean CC than the DT method under all transformations, while yielding better results for most of the effects used in testing relative to the ALTA method. For the occlusion effects, ALTA offered better results because it estimates the transformation matrix prior to matching. The points detected in the original image but not in the target image are ignored, whereas the SIFM method finds the correspondence of all detected points in the original image and predicts the point coordinates that are not detected in the target image.

To assess and compare the speed of different methods, the runtime per matching point and the response time was measured using SIFMDB dataset. The runtime achieved by different methods is presented in Table 1. The results indicate that the ALTA method was slightly faster than the proposed method in terms of average runtime, albeit at the expense of lower accuracy, as discussed previously. Based on the results presented in Table 1, DT method offers the highest average runtime. The DT method is also sensitive to the outliers resulting from the feature extraction method.

**Table 1:** Mean runtime for different methods under image effects.

	Rotation	U-Scale	Rot-Scale	Occlusion	Rot-Occ	Average
**SIFM**	1.44202	1.1132	1.45459	0.980164	1.475148	1.2930244
**ALTA**	0.98147	0.96578	1.35871	0.990158	1.294586	1.118141
**DT**	1.60245	1.51356	1.54896	1.124893	1.54879	1.467731

**Fig 6:**
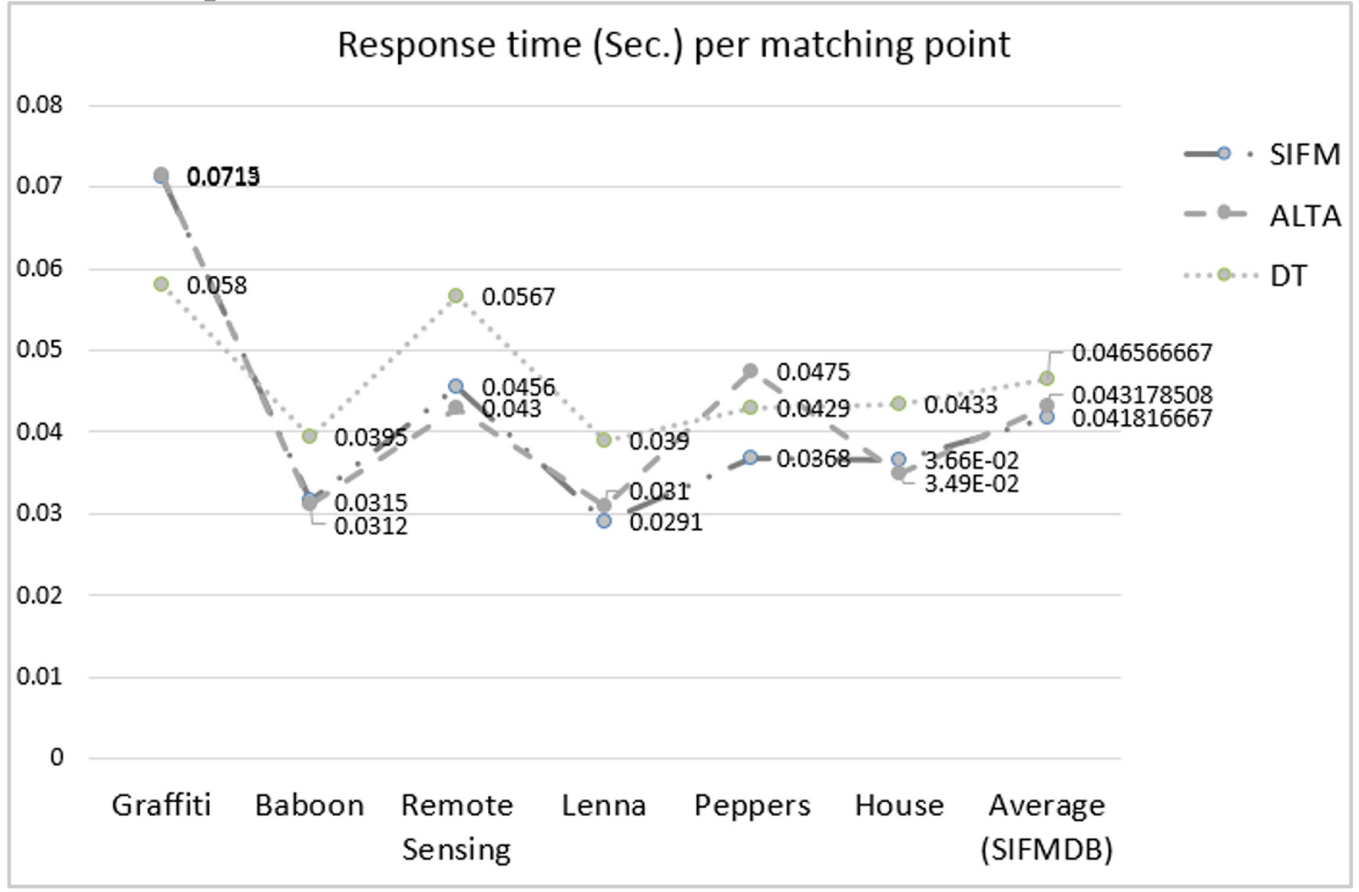
Average response time per matching point.

Different techniques achieved matching results using different number of detected points, which can affect their respective response times. Therefore, general response time presented in Table 1 is not a reliable indicator of the speed of different feature matching techniques. To address this problem, the runtime per matching points defined as *T*_*dp*_ is considered in this section. Considering the *T*_*o*_ as the total response time and *N*_*p*_ as the total number of detected points, the runtime per matching points can be defined as $T_{dp}=\frac{T_{o}}{N_{p}}$. The *T*_*dp*_ for different methods is presented in Fig 6.

**Fig 7:**
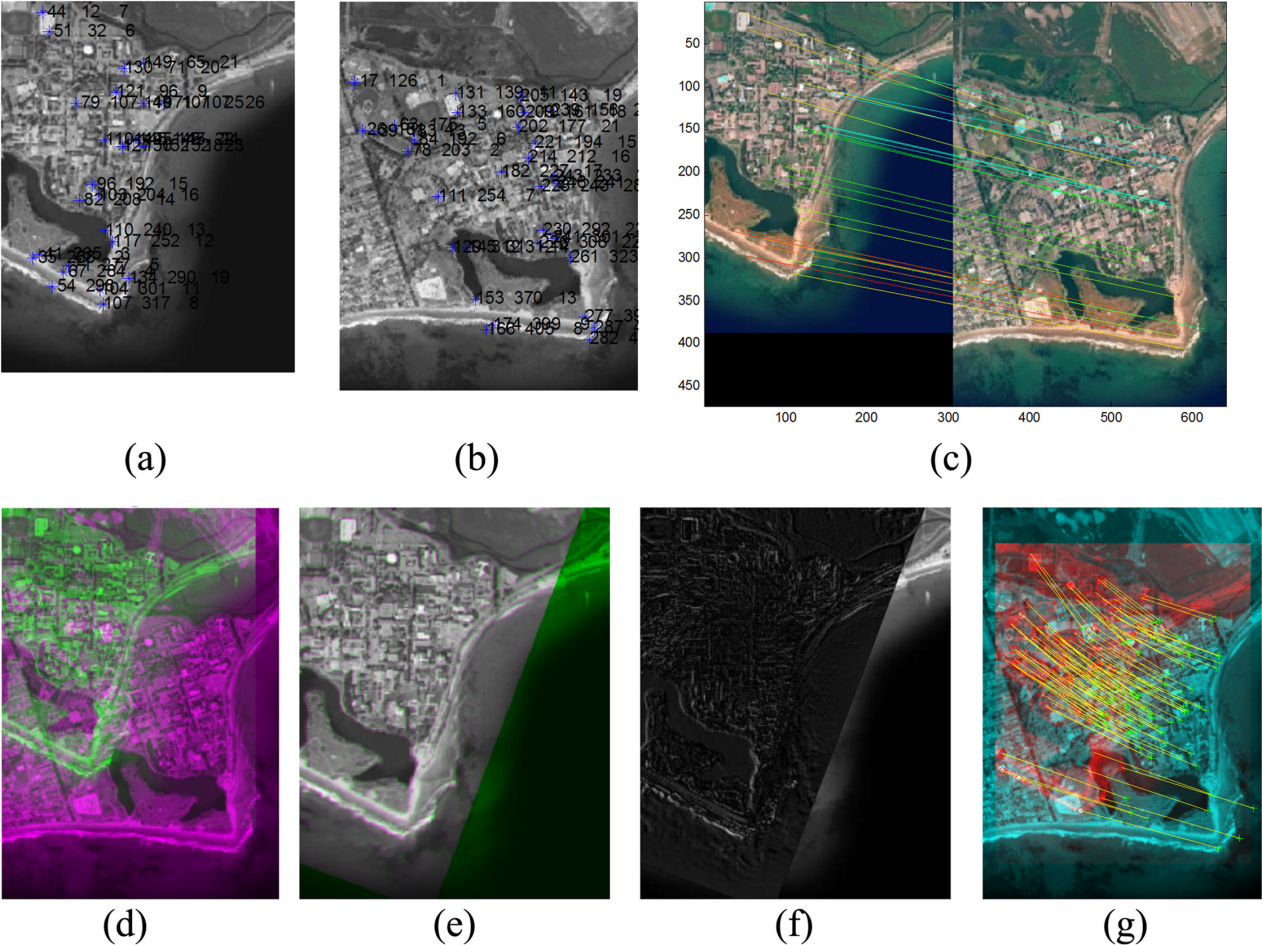
Image registration results of high resolution IKONOS satellite image (UC Santa Barbara) [40]. (a) Detected features on the source image; (b) Detected features on the target image; (c) Matching results; (d) Combination of source and target images before registration; (e) Combination of source and target images after registration; (f) Difference results after registration; (g) Feature movement after registration.

The response time per matching points results presented in Fig 6 indicate that, in some cases, ALTA achieved lower response time relative to other methods. The most accurate matched features were achieved by the proposed method; however, ALTA method also offers stable results under different effects. The proposed method increased the speed slightly and also improved the performance of the feature matching in terms of accuracy. On the other hand, DT method required higher runtime for most images and its matching accuracy is inadequate. In addition to the tests described above, the method proposed in this study was also used in an image registration application to demonstrate its robustness in a real word application. Image registration application is an important and frequently used application in different machine vision and image processing fields [44]. The results pertaining to the matched points extracted from the SIFM method can reconstruct the target image into the source image based on the information extracted from the transformation matrix in Eq (1). Fig 7 depicts the image registration results using the proposed technique.

**Fig 8:**
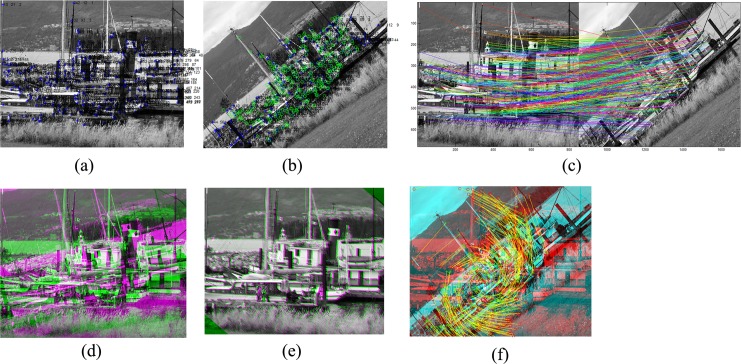
Image registration results of a highly deformed boat image (the Featurespace standard dataset) [32]. (a) Detected features on the source image; (b) Detected features on the target image; (c) Matching results; (d) Combination of source and target images before registration; (e) Combination of source and target images after registration; (f) Feature movement after registration.

Detected feature points in the source and target images are presented in Fig 7(A) and 7(B). The correspondence extraction results presented in Fig 7(C) indicate that most of the correspondence points are detected correctly; however, some correspondence points are incorrect or inaccurate. These inaccurate correspondence points do not significantly affect the final results, as estimating the transformation matrix eliminates the outliers in the registration stage. The overlapped image of the source and target images before and after registration process is presented in Fig 7(D) and 7(E), while Fig 7(F) presents the difference results, corresponding to the errors in the registration process. In the difference image, brighter pixels indicate points that are not registered correctly (errors), whereas darker pixels indicate points that are registered with higher accuracy. Fig 7(G) is provided to show the movement of the feature points during the matching process. To demonstrate the image registration results with high transformation effects, shown in Fig 8, boat image form the Featurespace standard dataset [32] is selected and is taken from different viewpoints.

Fig 8(A) and 8(B) present the detected feature points in the source and target images, respectively, while Fig 8(C) shows the matching results obtained by the proposed method. Fig 8(D) and 8(E) present the overlapped reference and target images before and after registration of the boat image, respectively. The matching results, including the movement of the points during the registration process, are shown in Fig 8(F).

To demonstrate the performance of the proposed method, six different image assessment techniques—Laplacian mean-square-error (LMSE), Peak Signal-To-Noise Ratio (PSNR), normalized cross-correlation (CC) [45], average differences (AD) [46], normalized absolute error (NAE) and Structural Similarity (SSIM) [47]—have been used. Table 2 presents the image registration results of different techniques, namely Multi-modality registration [48], Evolutionary strategy [49], Discrete Fourier [50], Coherent point drift [51] and the proposed method. The performance comparison provided in Table 2 indicates that the proposed method outperforms other evaluated techniques in terms of image quality assessment.

**Table 2:** Performance results for different image registration techniques applied to the Featurespace standard dataset [32].

Method	LMSE	PSNR	NCC	AD	NAE	SSIM
**Multi-modality registration [48]**	0.22127	54.6815	0.06157	0.38246	0.96902	0.00371
**Evolutionary strategy [49]**	0.19448	55.2420	0.21058	0.2957	0.8855	0.01836
**Discrete Fourier [50]**	0.09958	58.1488	0.79876	0.0067	0.59205	0.08344
**Coherent point drift [51]**	0.10099	58.087	0.7625	0.01076	0.5848	0.0732
**Proposed Method**	0.08276	58.9523	0.83284	0.02001	0.4051	0.20676

## Conclusions

A new invariant feature matching method is proposed for image registration application to overcome the limitations of the currently available techniques. The proposed method is based on extracting the information of triple features by relying on the dissimilarity value of the distinct path between two specific features. Two dissimilarity metrics known as NEC and SDD have been proposed to improve the accuracy of the feature matching technique. The proposed feature matching techniques utilize the most accurate correspondence points to estimate the transformation information of all feature points. Therefore, it is possible to predict the false negative features that were not detected in the first stage of matching. While the SIFM method is not only dependent on the local information of the features, it can extract the correspondence features with low localization accuracy. The evaluation results indicate that the SIFM method outperforms other methods in terms of average precision-recall, CC, and response time. However, it may fail when applied to images with a high degree of deformation changes or those with different modalities.

## Supporting Information

S1 Dataset SIFMDB dataset. (ZIP)
