# Supplementary figures and images for: Invariant Feature Matching for Image Registration Application Based on New Dissimilarity of Spatial Features
Source: PLoS One. 2016 Mar 17;11(3):e0149710. doi: 10.1371/journal.pone.0149710 (PMC4795769; doi:10.1371/journal.pone.0149710)

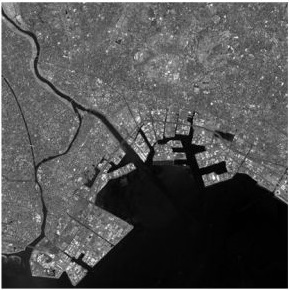

Supplement: S1 Dataset — (ZIP) [file pone.0149710.s001.zip › SIFMDB/666.jpg]

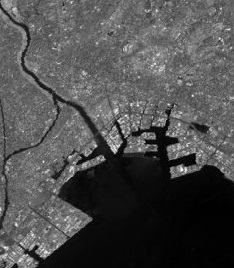

Supplement: S1 Dataset — (ZIP) [file pone.0149710.s001.zip › SIFMDB/666_1.jpg]

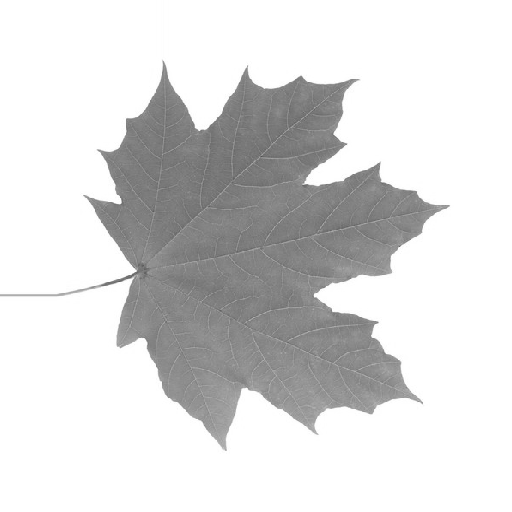

Supplement: S1 Dataset — (ZIP) [file pone.0149710.s001.zip › SIFMDB/acclaim002.bmp]

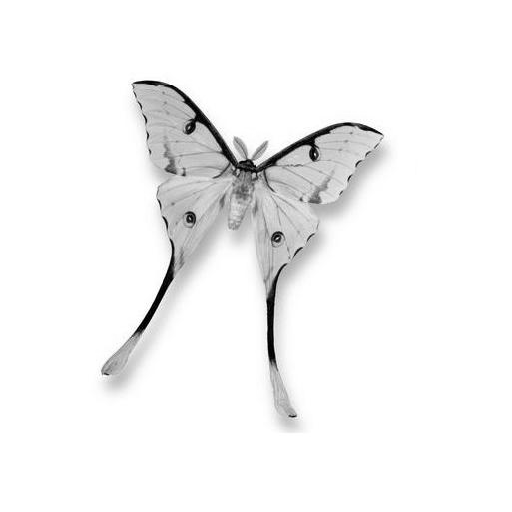

Supplement: S1 Dataset — (ZIP) [file pone.0149710.s001.zip › SIFMDB/acclaim005.bmp]

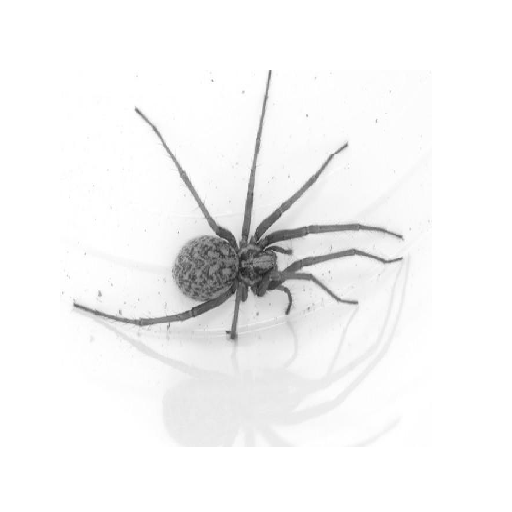

Supplement: S1 Dataset — (ZIP) [file pone.0149710.s001.zip › SIFMDB/acclaim006.bmp]

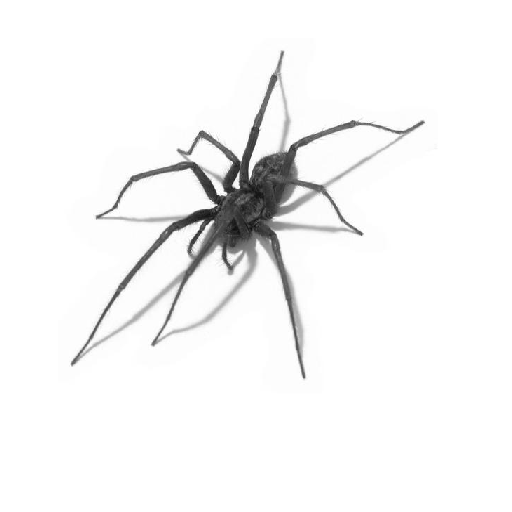

Supplement: S1 Dataset — (ZIP) [file pone.0149710.s001.zip › SIFMDB/acclaim007.bmp]

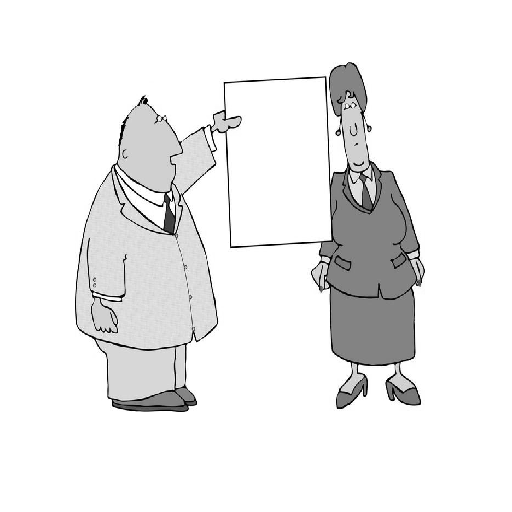

Supplement: S1 Dataset — (ZIP) [file pone.0149710.s001.zip › SIFMDB/acclaim008.bmp]

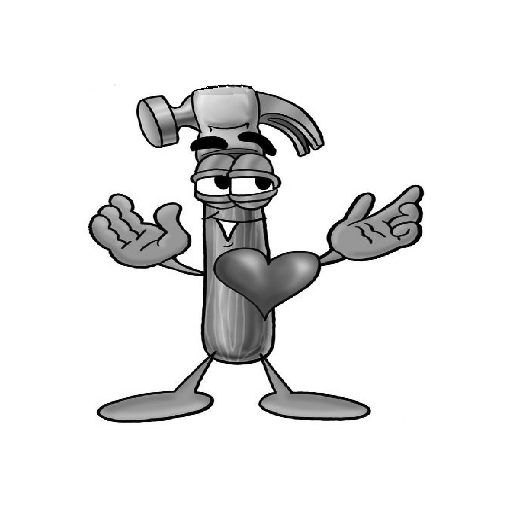

Supplement: S1 Dataset — (ZIP) [file pone.0149710.s001.zip › SIFMDB/acclaim009.bmp]

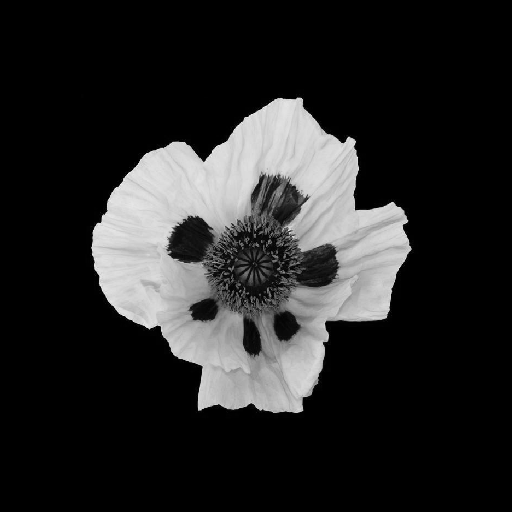

Supplement: S1 Dataset — (ZIP) [file pone.0149710.s001.zip › SIFMDB/acclaim010.bmp]

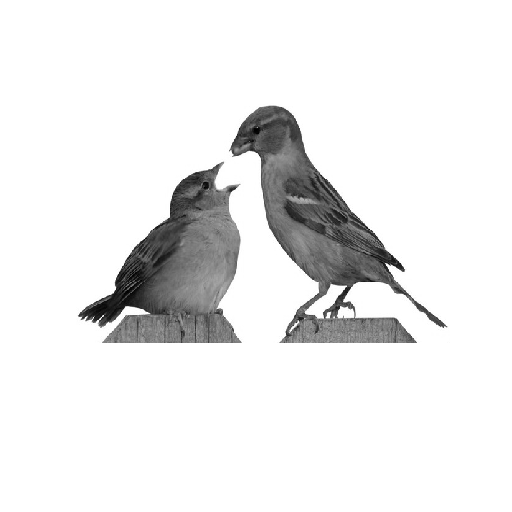

Supplement: S1 Dataset — (ZIP) [file pone.0149710.s001.zip › SIFMDB/acclaim012.bmp]

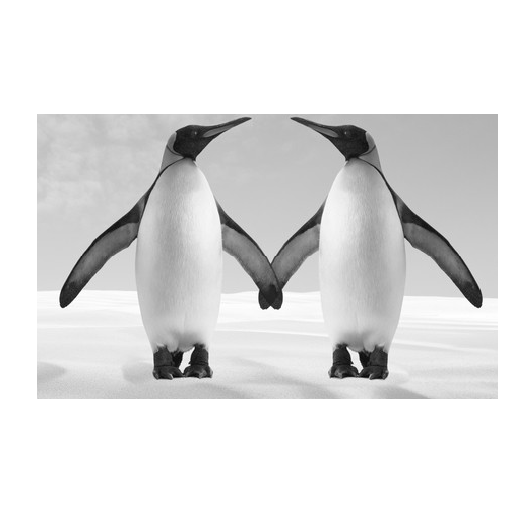

Supplement: S1 Dataset — (ZIP) [file pone.0149710.s001.zip › SIFMDB/acclaim013.bmp]

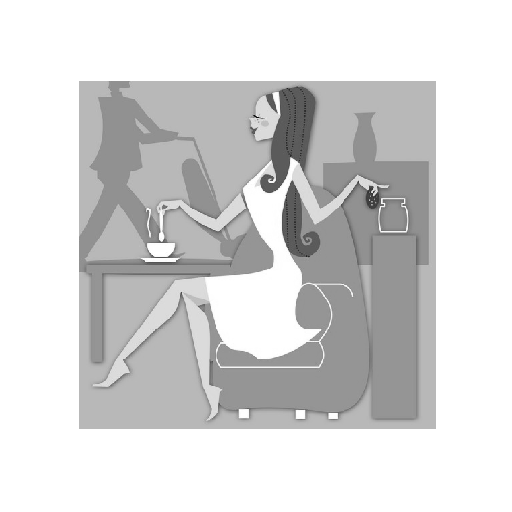

Supplement: S1 Dataset — (ZIP) [file pone.0149710.s001.zip › SIFMDB/acclaim014.bmp]

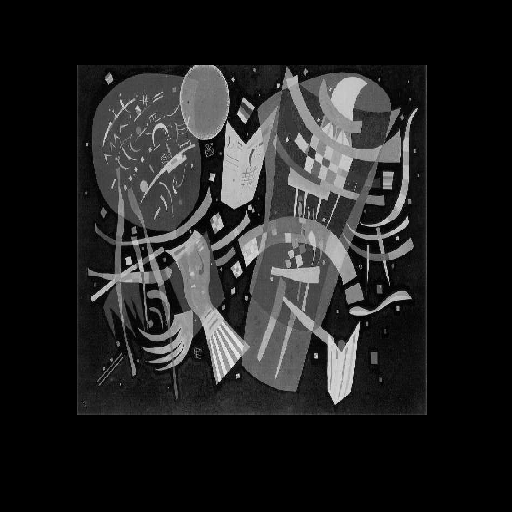

Supplement: S1 Dataset — (ZIP) [file pone.0149710.s001.zip › SIFMDB/art001.bmp]

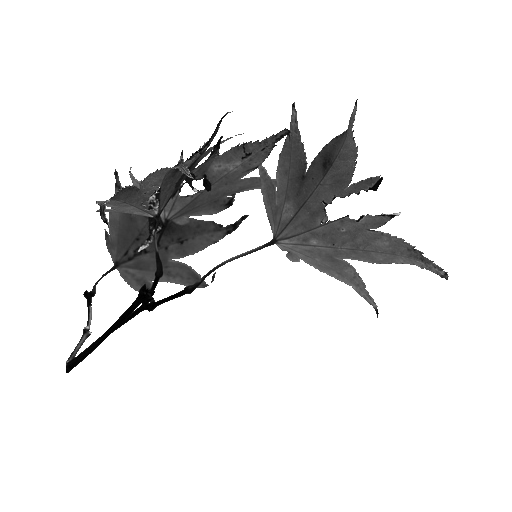

Supplement: S1 Dataset — (ZIP) [file pone.0149710.s001.zip › SIFMDB/autumn001.bmp]

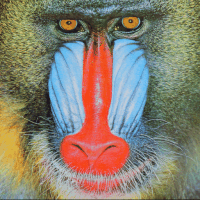

Supplement: S1 Dataset — (ZIP) [file pone.0149710.s001.zip › SIFMDB/Baboon.png]

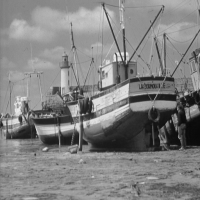

Supplement: S1 Dataset — (ZIP) [file pone.0149710.s001.zip › SIFMDB/boat.512.png]

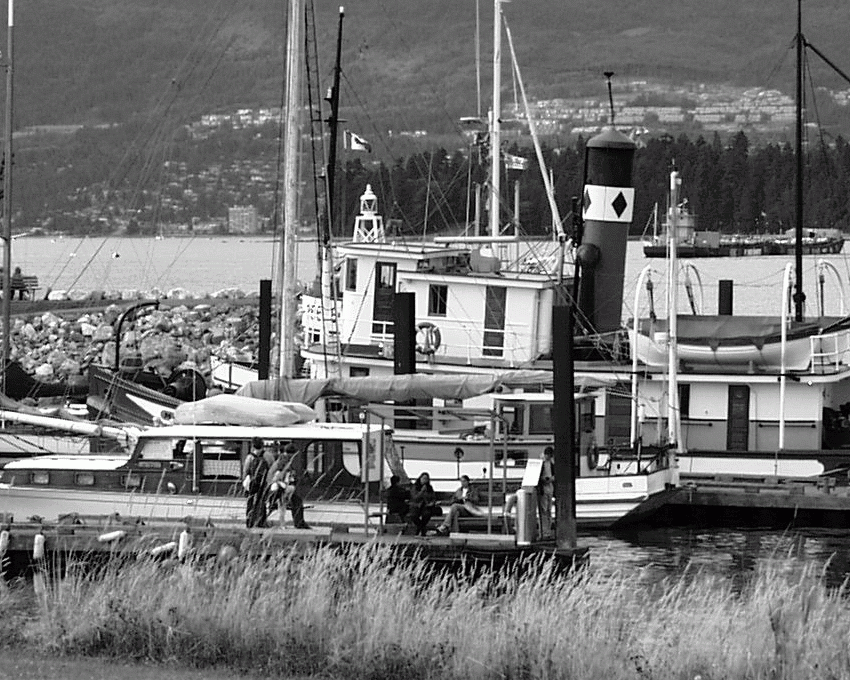

Supplement: S1 Dataset — (ZIP) [file pone.0149710.s001.zip › SIFMDB/boat1.png]

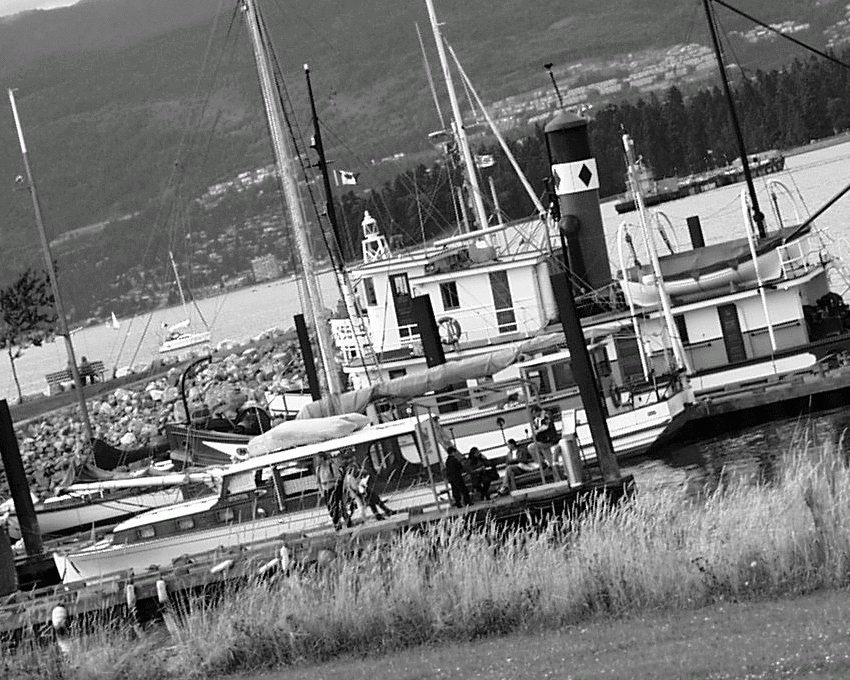

Supplement: S1 Dataset — (ZIP) [file pone.0149710.s001.zip › SIFMDB/boat2.png]

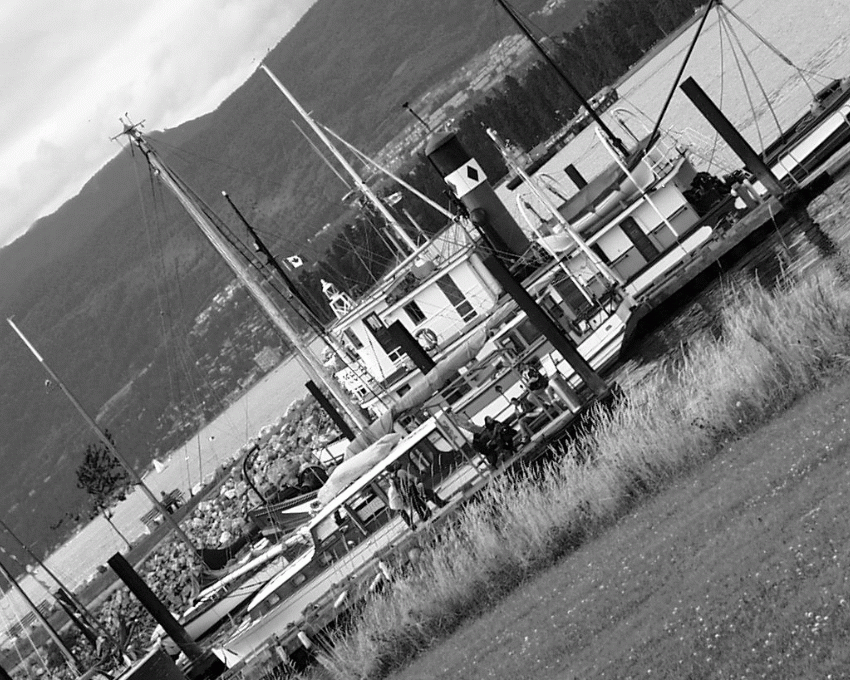

Supplement: S1 Dataset — (ZIP) [file pone.0149710.s001.zip › SIFMDB/boat3.png]

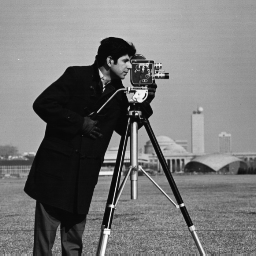

Supplement: S1 Dataset — (ZIP) [file pone.0149710.s001.zip › SIFMDB/Cameraman.jpg]

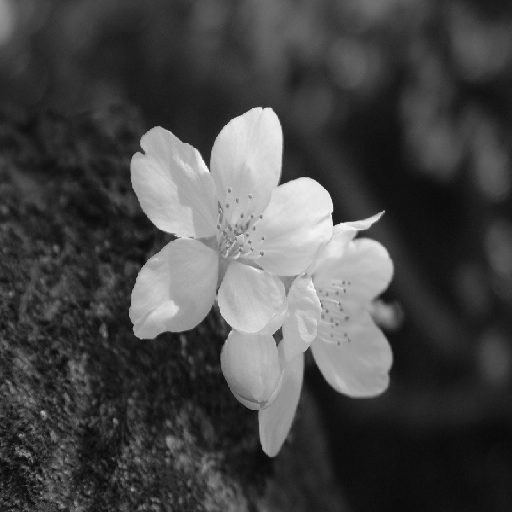

Supplement: S1 Dataset — (ZIP) [file pone.0149710.s001.zip › SIFMDB/flower001.bmp]

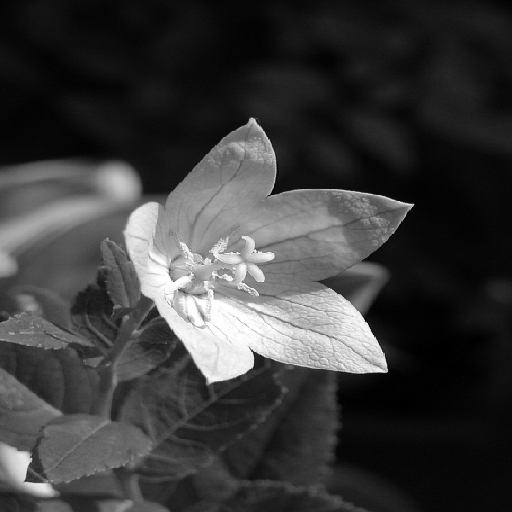

Supplement: S1 Dataset — (ZIP) [file pone.0149710.s001.zip › SIFMDB/flower002.bmp]

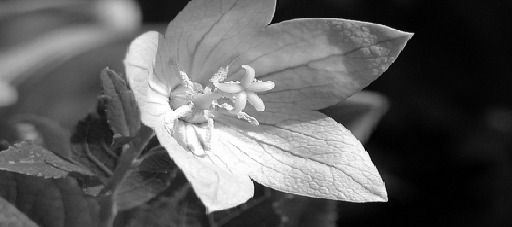

Supplement: S1 Dataset — (ZIP) [file pone.0149710.s001.zip › SIFMDB/flower002_cropped.bmp]

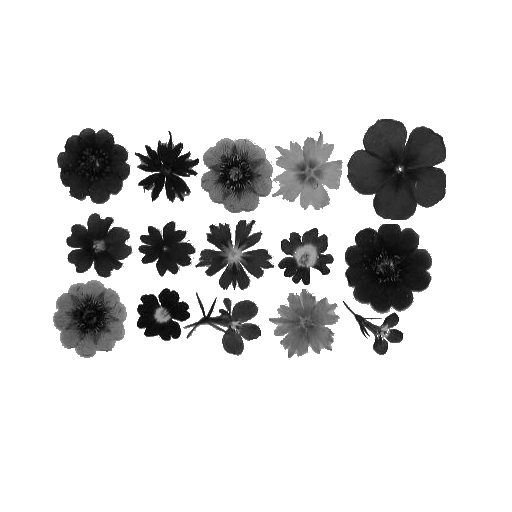

Supplement: S1 Dataset — (ZIP) [file pone.0149710.s001.zip › SIFMDB/flower003.bmp]

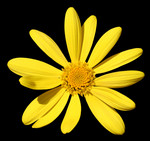

Supplement: S1 Dataset — (ZIP) [file pone.0149710.s001.zip › SIFMDB/flower1.jpg]

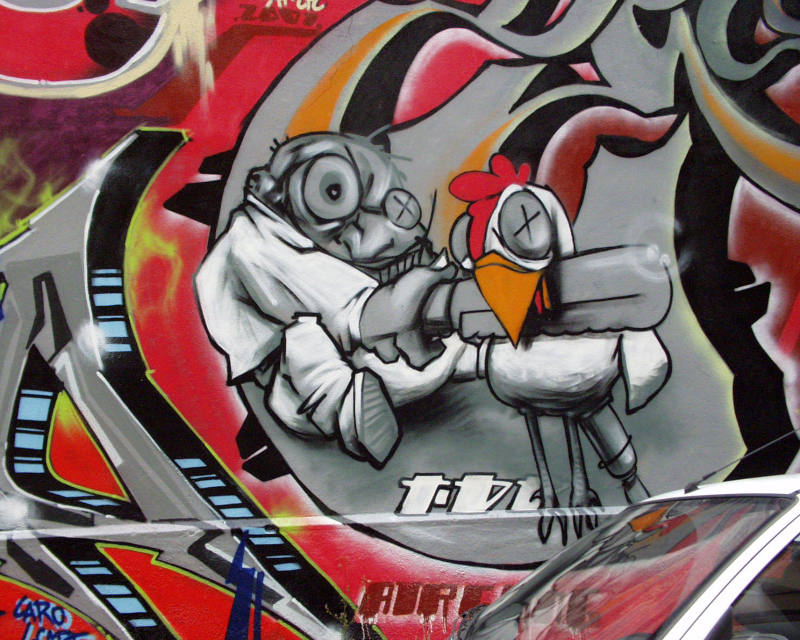

Supplement: S1 Dataset — (ZIP) [file pone.0149710.s001.zip › SIFMDB/graff1.png]

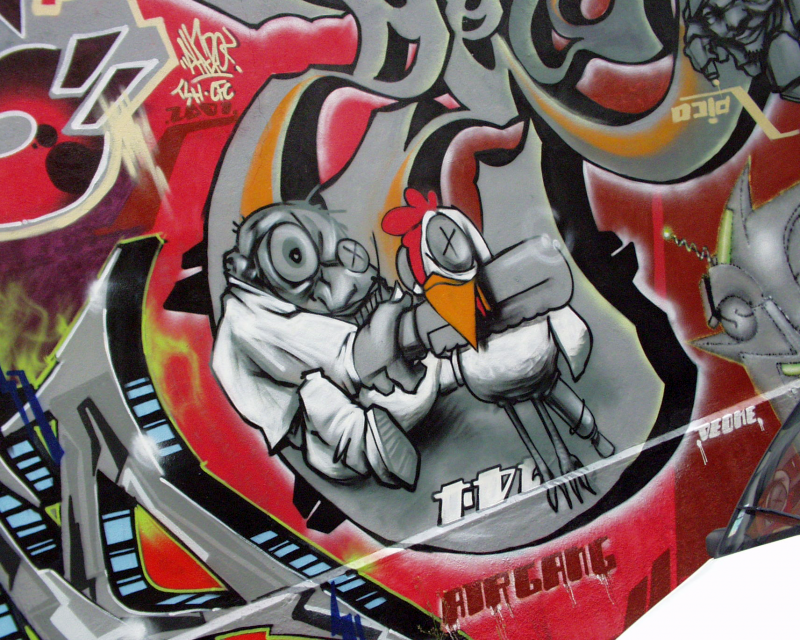

Supplement: S1 Dataset — (ZIP) [file pone.0149710.s001.zip › SIFMDB/graff2.png]

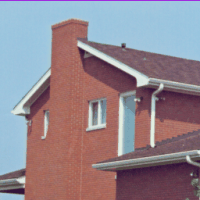

Supplement: S1 Dataset — (ZIP) [file pone.0149710.s001.zip › SIFMDB/House.png]

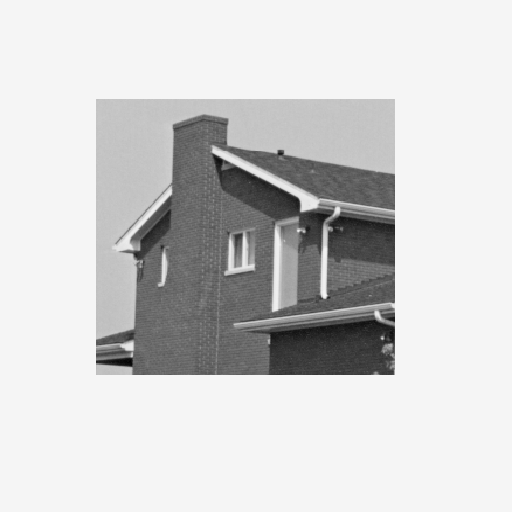

Supplement: S1 Dataset — (ZIP) [file pone.0149710.s001.zip › SIFMDB/house003.bmp]

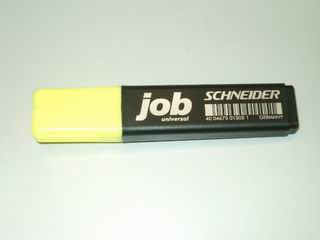

Supplement: S1 Dataset — (ZIP) [file pone.0149710.s001.zip › SIFMDB/image251.JPG]

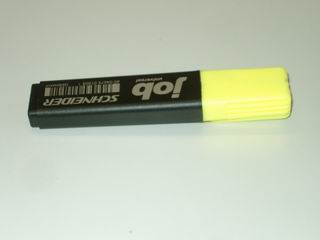

Supplement: S1 Dataset — (ZIP) [file pone.0149710.s001.zip › SIFMDB/image255.JPG]

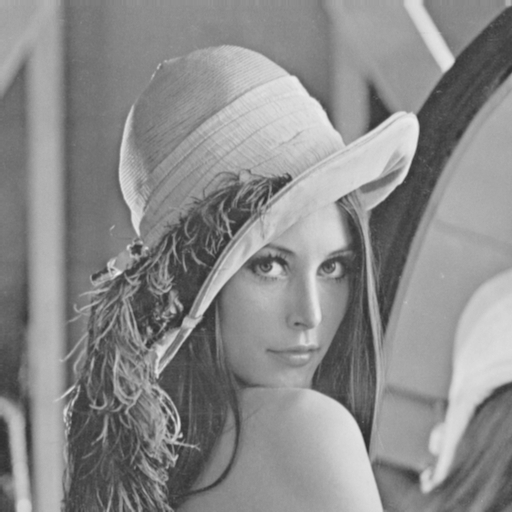

Supplement: S1 Dataset — (ZIP) [file pone.0149710.s001.zip › SIFMDB/Lenna.bmp]

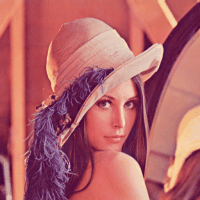

Supplement: S1 Dataset — (ZIP) [file pone.0149710.s001.zip › SIFMDB/Lenna.png]

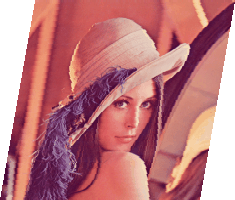

Supplement: S1 Dataset — (ZIP) [file pone.0149710.s001.zip › SIFMDB/LennaAffine.png]

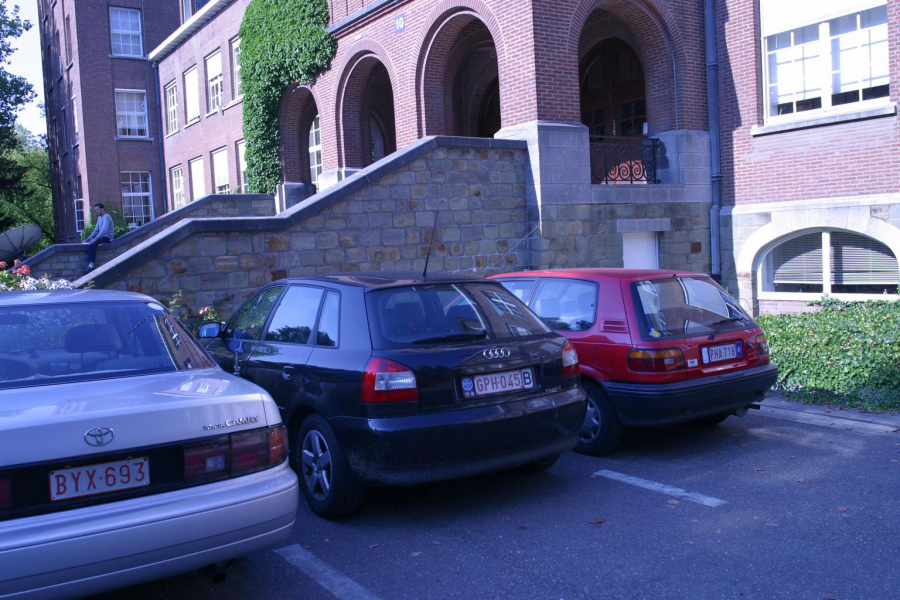

Supplement: S1 Dataset — (ZIP) [file pone.0149710.s001.zip › SIFMDB/light1.png]

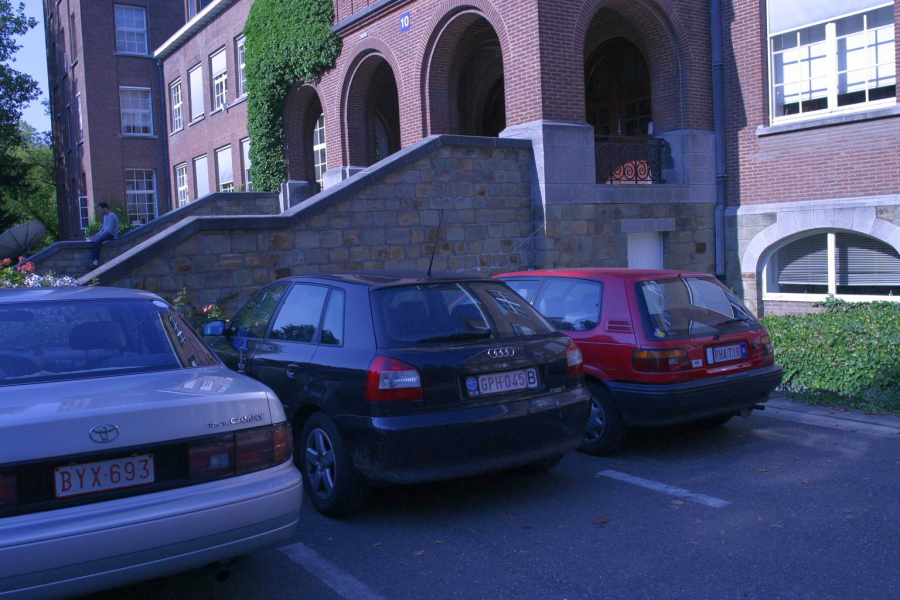

Supplement: S1 Dataset — (ZIP) [file pone.0149710.s001.zip › SIFMDB/light2.png]

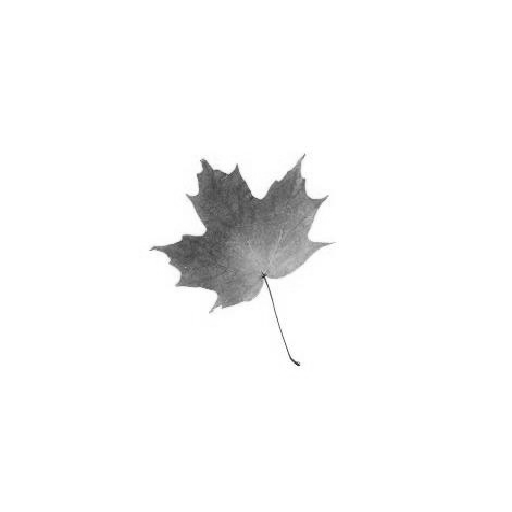

Supplement: S1 Dataset — (ZIP) [file pone.0149710.s001.zip › SIFMDB/maple-leaf001.bmp]

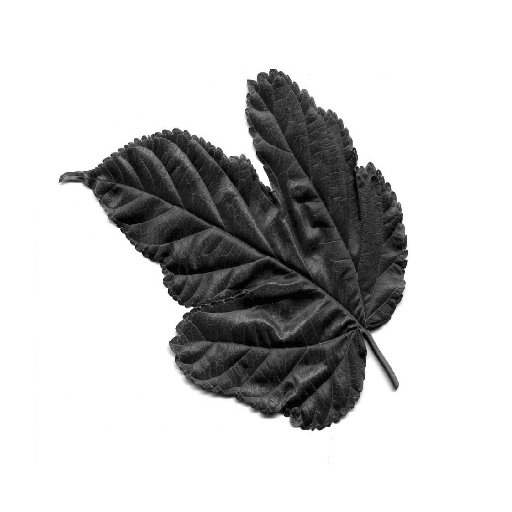

Supplement: S1 Dataset — (ZIP) [file pone.0149710.s001.zip › SIFMDB/maple-leaf002.bmp]

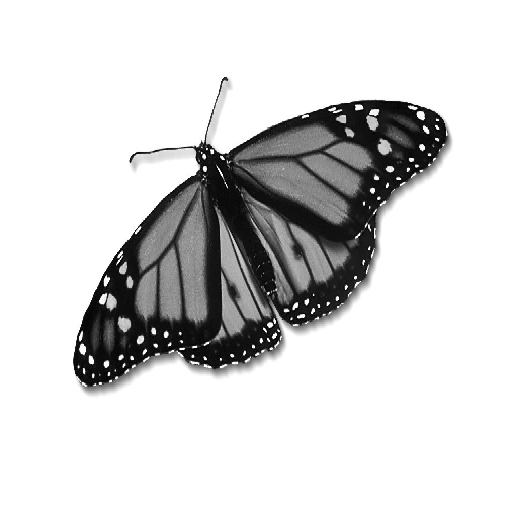

Supplement: S1 Dataset — (ZIP) [file pone.0149710.s001.zip › SIFMDB/monarch-butterfly.bmp]

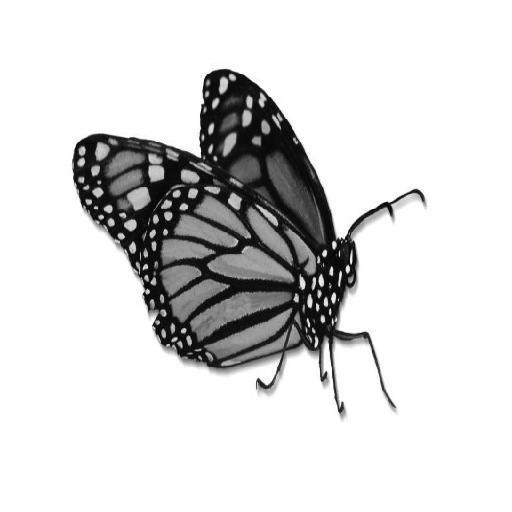

Supplement: S1 Dataset — (ZIP) [file pone.0149710.s001.zip › SIFMDB/monarch-butterflyside.bmp]

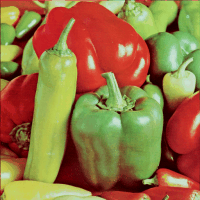

Supplement: S1 Dataset — (ZIP) [file pone.0149710.s001.zip › SIFMDB/Peppers.png]

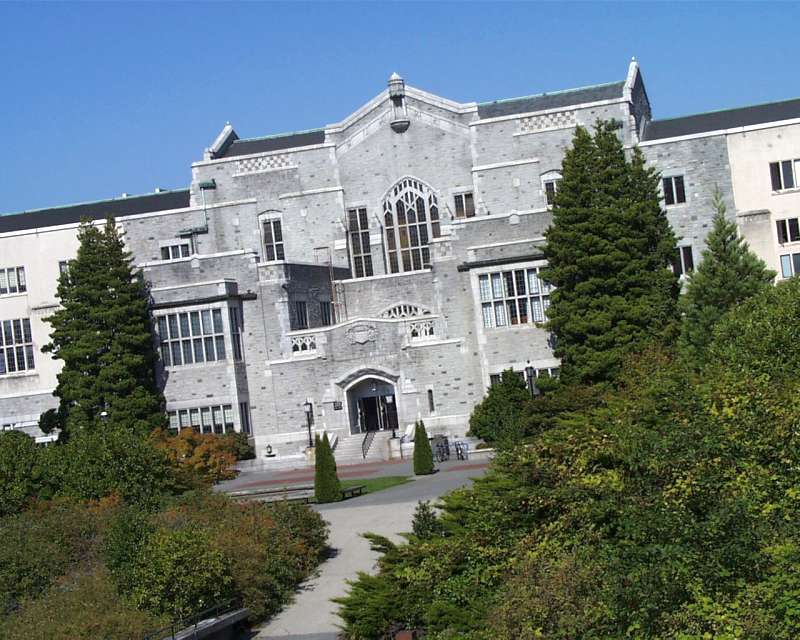

Supplement: S1 Dataset — (ZIP) [file pone.0149710.s001.zip › SIFMDB/ubc1.png]

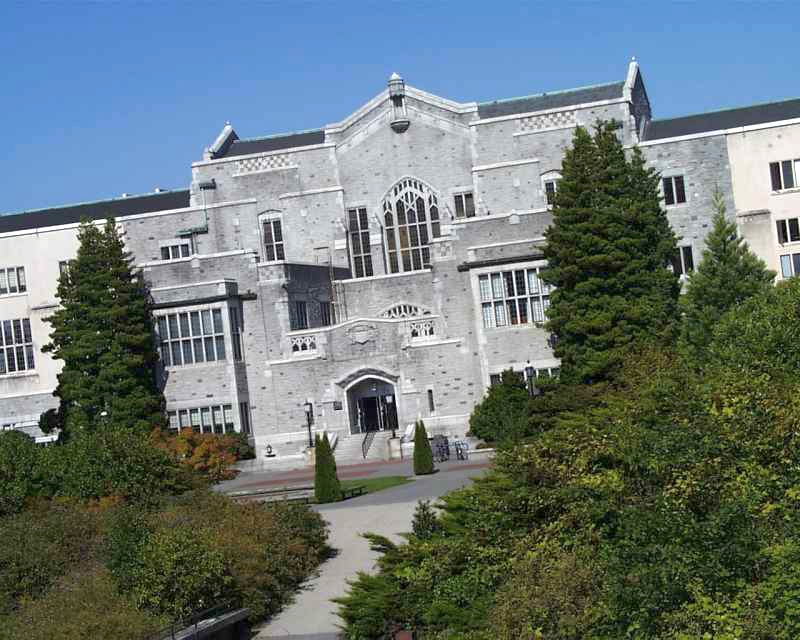

Supplement: S1 Dataset — (ZIP) [file pone.0149710.s001.zip › SIFMDB/ubc2.png]

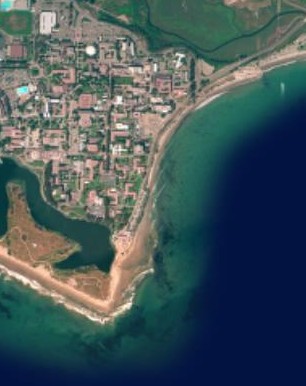

Supplement: S1 Dataset — (ZIP) [file pone.0149710.s001.zip › SIFMDB/ucsb1.jpg]

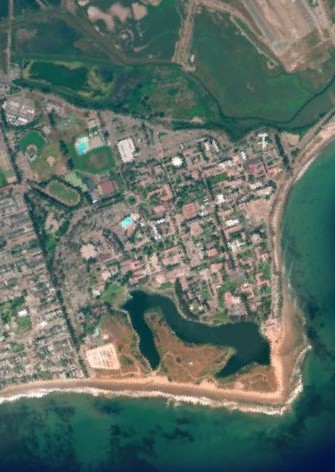

Supplement: S1 Dataset — (ZIP) [file pone.0149710.s001.zip › SIFMDB/ucsb2.jpg]

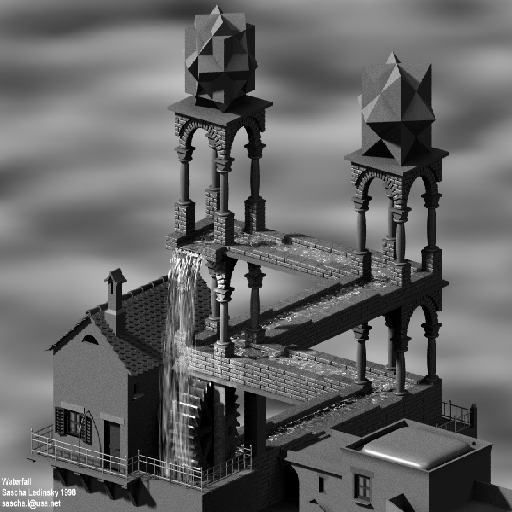

Supplement: S1 Dataset — (ZIP) [file pone.0149710.s001.zip › SIFMDB/waterfall.bmp]
